# Urban Land Use Decouples Plant-Herbivore-Parasitoid Interactions at Multiple Spatial Scales

**DOI:** 10.1371/journal.pone.0102127

**Published:** 2014-07-14

**Authors:** Amanda E. Nelson, Andrew A. Forbes

**Affiliations:** Department of Biology, University of Iowa, Iowa City, Iowa, United States of America; University of Iowa, United States of America

## Abstract

Intense urban and agricultural development alters habitats, increases fragmentation, and may decouple trophic interactions if plants or animals cannot disperse to needed resources. Specialist insects represent a substantial proportion of global biodiversity and their fidelity to discrete microhabitats provides a powerful framework for investigating organismal responses to human land use. We sampled site occupancy and densities for two plant-herbivore-parasitoid systems from 250 sites across a 360 km^2^ urban/agricultural landscape to ask whether and how human development decouples interactions between trophic levels. We compared patterns of site occupancy, host plant density, herbivory and parasitism rates of insects at two trophic levels with respect to landcover at multiple spatial scales. Geospatial analyses were used to identify landcover characters predictive of insect distributions. We found that herbivorous insect densities were decoupled from host tree densities in urban landcover types at several spatial scales. This effect was amplified for the third trophic level in one of the two insect systems: despite being abundant regionally, a parasitoid species was absent from all urban/suburban landcover even where its herbivore host was common. Our results indicate that human land use patterns limit distributions of specialist insects. Dispersal constraints associated with urban built development are specifically implicated as a limiting factor.

## Introduction

Global intensification of urban and agricultural land use has critical implications for biological diversity. Insects provide ecosystem services (including pest control, pollination, waste decomposition, and a food source for other important species) worth an estimated $57 billion annually in the U.S. [1]. Conservation of species providing these services requires an understanding of both the spatial scale of service provision and the dynamics of insect movements across landscapes [2]. Intensive land use has generally been associated with declines in biodiversity [3], but more nuanced relationships between differing degrees of land use intensity and insect biodiversity are not well characterized, particularly across spatially heterogeneous landscapes [4]. Spatial structuring of plant habitats and characteristics of the intervening matrix between habitats have important consequences for distributions of natural enemies [5], [6]. As intensity of development increases on a gradient from natural systems to agricultural land to urban landcover, patches of suitable habitat tend to become smaller in area and increasingly isolated [7]. Agricultural landcover often contains inadequate spatial and temporal plant resource diversity (i.e. food, habitat) to sustain diversity at higher trophic levels [8]. In cities, plant resources tend to be highly diverse but configured in complex mosaics of disparate habitat types often characterized by frequent disturbance as well as altered biogeochemical, hydrological, and temperature regimes [9].

Insects comprise a large proportion of animal diversity, and many of these are phytophagous specialists [10]. Distributions and diversity of plants in complex landscapes may therefore have substantial impacts on insect diversity if many insects interacting across two or more trophic levels are host specialists. Indeed, landscape level plant diversity strongly affects phytophagous insect distributions—population establishment depends on the presence of appropriate hosts [11]. However, spatially explicit studies that consider impacts of the plant community on insect diversity across both primary and secondary consumer levels remain rare (but see [12], [13], [14]).

Constraints associated with body size, trophic level, and specialist insect life histories may influence sensitivity to landscape alteration by humans. Because dispersal distances are often positively correlated with body size, actively dispersing insects are expected to respond to landcover patterns at finer spatial scales as body sizes decrease [5]. Predators generally experience landscapes at a larger spatial scale than herbivores [15]; however, because most parasitoid wasps (Hymenoptera that fatally parasitize other insect hosts) are smaller in size than their hosts, these natural enemies may have lower dispersal abilities and smaller effective spatial scales than their hosts [16]. Thus, while specialist insect herbivores may be impacted by land use patterns, impacts may be even greater for their specialist parasitoids. Indeed, intensive land use has been shown to disrupt trophic interactions between herbivores and parasites in both urban [6] and agricultural [16] contexts.

In this study, we used a spatially explicit framework to investigate tri-trophic species interactions across a complex, human-altered landscape. Specifically, we investigated the degree to which plant-herbivore-parasitoid interactions are decoupled across varying intensities of urban and agricultural development at varying spatial scales of analysis. Our study landscape in the Midwestern US is more than 90% human-altered, thus representing an extreme case reflective of global agricultural and urban land use trends [17]. We focused on two tree species native to North America, each of which is the exclusive host of a *Rhagoletis* fruit fly species, and each of which is in turn attacked by specialist parasitoid wasp species. Inter-trophic specialization facilitates predictions regarding population-level responses to landscape architecture because it represents the most simplified model of food web structure [6], [18]. Both tree-fly-wasp systems are characterized by strong host fidelity across all trophic levels [19], [20], enabling direct assessment of the extent of inter-trophic decoupling (i.e. disruption or elimination of one trophic level's tendency to co-occur with the tropic level directly below). We hypothesized that built human landcover would negatively impact insect presence and density, and that this effect would become stronger with increasing trophic level. Specifically, we predicted that trophic interactions would be more strongly decoupled in increasingly intense urban development relative to agricultural development. Further, we predicted that parasitoid wasps would be more sensitive to effects of land use than herbivorous flies.

## Methods

No specific permits were required for this study. The study sites were a combination of private and publicly held lands. Contacts for sampling on public lands included the Iowa City Parks and Recreation Division and the Johnson County, IA Secondary Roads Department. Permissions to be on private land were obtained from individual landowners. No protected species were sampled.

### Study Organisms

Our study organisms include two tri-trophic (tree-fly-wasp) systems. Black cherry (*Prunus serotina* Ehrh. [Rosales: Rosaceae]) and black walnut (*Juglans nigra* L. [Juglandales: Juglandaceae]) host the cherry fruit fly, *R. cingulata* Loew, and the walnut husk fly, *R. suavis* Loew (Diptera: Tephritidae), respectively. In this study, we also focus on a parasitoid of the cherry fly, *Diachasma ferrugineum* Gahan (Hymenoptera: Braconidae), and a parasitoid of the walnut fly, *Coptera pomonellae* Muesebeck (Hymenoptera: Diapriidae). All insects are univoltine and only adult stages are capable of dispersal.

Black cherry and black walnut are both native North American trees with seeds enclosed in fleshy fruit tissue. *Prunus serotina* is an insect-pollinated, shade-intolerant, secondary successional species that occupies fencerows and edges of mesic forest in the southeastern two-thirds of North America [21]. *Juglans nigra* is wind-pollinated, occupies approximately the same geographic area, is equally shade-intolerant, and principally occupies edges of mesic forest but may also thrive singly in open (herbaceous) patches [22]. In the Midwestern United States, black cherry fruits ripen from June through September, while black walnut fruits ripen from September through early November. Both tree species begin fruiting when young (>5 y), reach maximum fruit production after 30 years, and may ultimately live to be over 100 years old.

*Rhagoletis* (Diptera: Tephritidae) flies mate on or near their host tree and lay eggs in ripening fruits. Larval instars 1 through 3 feed on fruit pulp. When fruits abscise, late third instar larvae exit the fruit and pupate in the top ∼10 cm of soil [23]. Adult flies eclose the following year and use visual and olfactory cues to locate their host fruits [24]. While *R. cingulata* lay one egg per black cherry fruit, *R. suavis* flies lay clutches of up to 100 eggs in a single walnut fruit. Larval aggregation within walnut fruits due to the gregarious oviposition strategy of *R. suavis* can be further compounded by superparasitism, wherein two or more females lay egg clutches in the same fruit [25].

Parasitoid wasps of *Rhagoletis* attack egg, larval, or pupal stage flies. In this study, we focus on *Diachasma ferrugineum* Gahan (Hymenoptera: Braconidae), a larval parasitoid of *R. cingulata*, and *Coptera pomonellae* Muesebeck (Hymenoptera: Diapriidae), a pupal parasitoid of *R. suavis*. *Diachasma ferrugineum* females oviposit into late instar *R. cingulata* larvae feeding inside *P. serotina* fruits [26]. *Diachasma* wasps orient to their fly hosts via visual and olfactory cues associated with fruits and also respond to mechanical stimuli from fly larvae moving in fruits [27; reviewed in 28]. The cherry fly is also host to three additional parasitoids: another larval parasitoid, *Diachasmimorpha mellea* Gahan (Hymenoptera: Braconidae); an egg parasitoid, *Utetes frequens* Fischer (Hymenoptera: Braconidae); and a pupal parasitoid, *Coptera cingulatae* Muesebeck (Hymenoptera: Diapriidae). The cherry flies in this study were only collected in their larval stages, so we did not rear any *C. cingulatae* in this study. The other two parasitoids of the cherry fly we found only rarely; we reared a total of five *Diachasmimorpha mellea* and seven *U. frequens* from a total of five sites. *Coptera pomonellae* is the only recorded parasitoid of *R. suavis*. *Coptera* identify their hosts in soil by sensing trails from rotting fruit liquid and exudates emitted by fly larvae [29]. Female *C. pomonellae* have modified shovel-shaped heads that allow them to burrow in soil to locate and oviposit into fly pupae [30].

### Data Collection

**Figure 1:**
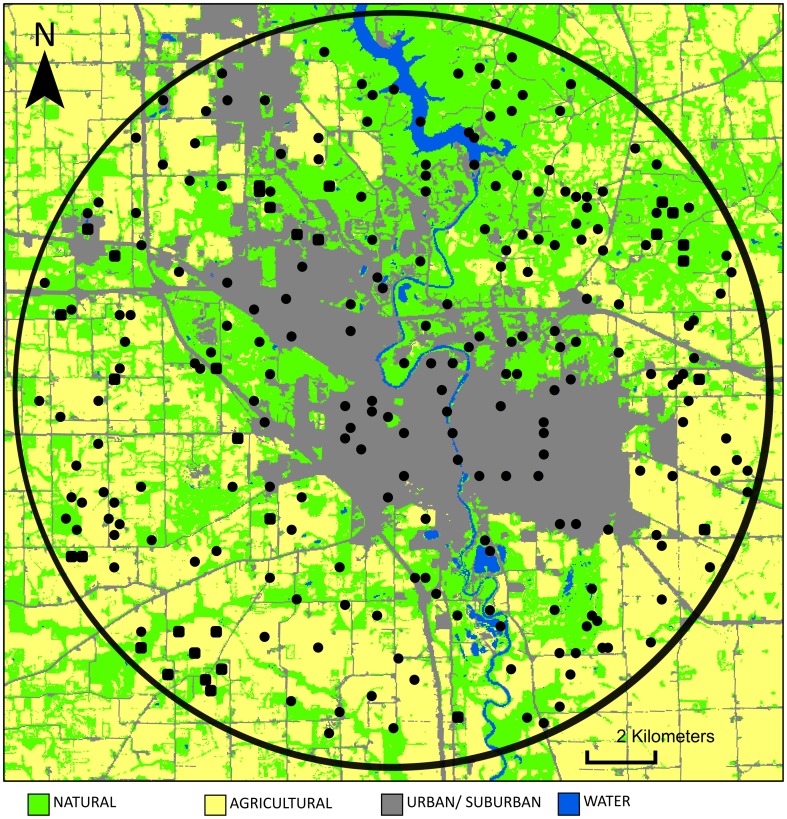
250 study sites. Sites (black dots) were 150×150 m in size and were randomly selected from a 360 km^2^ landscape (black circle) in Johnson County, IA, USA. All sites were inventoried for presence and abundance of black walnut and black cherry trees and their respective fruit flies and parasitoids (see methods). Landscape areas shown include urban/suburban development (gray), agricultural land (yellow), “natural” wooded and herbaceous areas (green), and water (blue).

We used aerial photographs (taken in 2010) in ArcInfo (ESRI, Redlands, CA) to select a 360 km^2^ study area in Johnson County, Iowa, USA that contained extensive urban/suburban development and agricultural landcover. This study area was digitally rendered and divided into 15,188 individual plots, each 150×150 m in size. From these, we selected 250 sampling sites (Figure 1) using a non-repetitive random number generator so that the proportion of sites occupied by taxa could be extrapolated to landscape level trends.

We visited 248 study sites in May 2011 to inventory each for presence and density of black walnut and black cherry trees (two aquatic sites were excluded from our analyses). Tree density per site area was quantified by counting all sexually mature trees within site boundaries determined using 2010 aerial photos with 20 cm resolution. Aerial photos were also used as a reference to ensure that the entire site area was assessed for walnut and cherry trees. We surveyed heavily wooded sites using a handheld global positioning system (GPS) (Garmin eTrex Venture, Garmin International Inc., Olathe, KS) to track our survey paths within superimposed site boundaries because aerial photos did not provide adequate reference points in these areas. On all sites, black walnut and black cherry tree locations and numbers were marked with GPS waypoints. The resulting point data was overlain upon aerial photos in ArcInfo for visual reference when collecting fruits.

We returned in July-November 2011 to collect walnut and/or cherry fruits from all sites containing trees. We estimated fruit abundances for both tree species at each site via agreement between two or more independent observers upon the proportion of fruits collected relative to total fruits present; estimates were then calculated for entire sites based on counts of collected fruits. Ripe fruits of both tree species were collected from all fruiting trees across each site. We haphazardly sampled fruits from trees, approximately in proportion to fruit abundance (i.e. trees with more fruits were sampled more heavily). Fruit collections were limited to ≤50% of fruits per site to avoid collateral impacts on insect population dynamics. Black walnut fruits do not change in appearance when ripe and were therefore collected only after they had abscised from trees. Black cherry fruits change markedly from green to red to black when ripe. Ripe black fruits were collected haphazardly from various heights across cherry trees (up to a maximum of 6 m) using several methods including manually picking fruits, shaking branches over tarps, and cutting high racemes with a 4 m telescoping pruner while on a ladder. On two cherry sites, where individual trees were sufficiently tall that we could not reach their lowest branches, we collected freshly fallen fruits from the ground.

Because larval insects are found within fruits, a sampling of ripe fruits equates to sampling the lower two trophic levels in the walnut system (tree and fly) and all three trophic levels in the cherry system (tree, fly, and wasp). Collected fruits were held in wire mesh baskets over greenhouse plant starter trays. We counted emerging fly larvae, placed them in petri dishes with moist vermiculite until they pupated, and artificially overwintered pupae for 8 months at 4°C. Because the walnut wasp attacks flies only after they pupate in soils, we also performed collections of fly pupae from soils beneath walnut trees at a limited (haphazardly selected) subsample of sites (11/54). On each of these sites, we used spades to loosen soil under fallen walnut fruits at several (5–10) locations under each fruiting tree on each site and manually sifted the soil for *R. suavis* pupae. Pupae were then covered lightly in soil and returned to the laboratory to be counted and placed in vermiculite. Subsampling was carried out for walnut wasps due to seasonal time constraints. Soil collected pupae were overwintered in the manner described above. Following overwintering, we gradually warmed insects to 25°C with a 12∶12 h light: dark cycle to induce breaking of diapause. Emerging adult flies and parasitoid wasps were identified to species and counted.

### Geoprocessing and Categorization of Sites

We quantified landcover at 30 m resolution in ArcInfo from the 2011 USDA CropScape data layer [31], which uses maximum likelihood methods to categorize landcover from reflectance values in Landsat Thematic Mapper data (ground-truthing in Iowa showed 94.6% accuracy for this data) [31]. Following Cushman and Landguth [32], we define a spatial scale as a unique combination of thematic resolution, grain, and extent. “Thematic resolution” refers to the level of ecological detail captured, “grain” refers to the resolution with which geographic detail is captured, and “extent” is the area (in m^2^) of land sampled. In order to investigate associations between land use and trophic interactions at a wide range of spatial scales, we categorized sites and the areas around them at two thematic resolutions (site occupancy and herbivory/parasitism rates), at two grains (coarse and fine grain landcover), and for six extents (specific buffer radii around plot centroids, see below).

From 20 landcover classes delineated in the CropScape dataset for our study area, we grouped sites and landcover polygons into fine- and coarse-grain categories (Table 1). The distinction between coarse and fine grain refers to differences in both thematic and spatial resolution. For categories at coarse grain resolution, sites were assigned to either “urban/suburban development”, “agricultural development”, or “natural” (wooded and herbaceous areas) according to the dominant landcover within 3 km surrounding each site in ArcInfo at a 1∶100,000 scale and 150 m spatial resolution. This buffer distance was used for consistency in site classification; the majority of sites are embedded in the same coarse grain category judged over a broad range of distances (from the site level to ∼5 km). Three km also represents a liberal estimate of maximum dispersal distance for *Rhagoletis* flies [33]. For five sites at the interface of agricultural and city landcover the dominant category was unclear at a 3 km distance, so we categorized these according to dominant landcover at a 1 km distance. We assigned fine grain categories within individual (150×150 m) sites according to the landcover class occupying >50% of the area. Fine grain categories were captured with a higher degree of both geographic and thematic detail. Note that coarse and fine grain categories were assigned independently of one another (e.g. wooded cover at the plot level was present in all three coarse grain, landscape level categories). Fine grain composition of coarse grain landcover categories is shown in Figure S1. This scheme resulted in classification of sites according to the type of landcover in which host trees were locally embedded. Ground-truthing was performed on sites to ensure that fine grain categories were accurate. We also quantified proportions of fine grain landcover (similar to [34]) in progressively larger radii surrounding the centroid of each plot at 250 m, 500 m, 1 km, 2 km, 3 km, and 4 km, respectively (see Appendix S1 for geoprocessing details). This latter method allowed us to assess the importance of landcover composition with a level of detail that might be lost in a process of strict categorization by dominant landcover on sites.

**Table 1:** Coarse- and fine-grain landscape categories used in this study.

Coarse Grain Categories	Fine Grain Categories	CropScape Categories
Urban/Suburban development	High density development (HDD)	High intensity developed
		Barren
	Medium density development (MDD)	Medium intensity developed
	Low density development (LDD)	Low intensity developed
	Open development (OD)	Open development
Agricultural Development	Cropland (CROP)*	Soybeans (IPC)
		Alfalfa (IPC)
		Clover (IPC)
		Corn (WPC)
		Oats (WPC)
		Rye (WPC)
		Spring & winter wheat (WPC)
		Switchgrass (WPC)
“Natural”	Wooded	Deciduous forest
		Woody wetlands
		Shrubland
	Herbaceous (HERB.)	Grassland herbaceous
		Herbaceous wetlands
NA	Water	Open water

Landcover types were delineated based on 30 m resolution CropScape data (USDA-NASS, 2011). Abbreviations used in reporting of fine grain results are shown in parenthesis. Urban/suburban categories are listed in order of decreasing density of impervious surface; “open development” consists primarily of mowed grass and roads. *Cropland was divided into insect- (IPC) versus wind-pollinated crops (WPC) for regression analyses.

### Data Analysis: Occupancy and Density

We compared site occupancy and organismal densities on sites in coarse and fine grain landcover categories within and between trophic levels to assess the extent to which trophic decoupling was observed with more intense landcover and higher trophic level. We compared proportions of sites occupied by trees, flies, and wasps within species in both systems across coarse and fine grain categories using pairwise Fisher's exact tests. Variation in site occupancy patterns with coarse and fine landcover were then compared graphically for congruence across trophic levels within each plant-herbivore-parasitoid system. We also performed Chi-squared tests on data matrices designed to directly test the hypothesis that occupancy patterns across trophic levels differ with landcover among fine and coarse grain categories. We divided our occupancy data into four categories, coded as: 0-0-0 (no trees, flies, or wasps), 1-0-0 (trees, but no flies or wasps), 1-1-0 (trees and flies, but no wasps), and 1-1-1 (trees, flies, and wasps). We then tested for significant deviations from a null expectation: that counts of the four possible tri-trophic occupancy outcomes would be proportional among landcover categories.

We performed Kruskal-Wallis and Mann Whitney U-tests in SPSS (IBM, Armonk, NY) to compare tree densities and rates of herbivory/parasitism within insect species for coarse and fine grain landcover categories. These results were likewise compared graphically across trophic levels to assess the extent of congruence in density patterns with respect to landcover. We also used Kruskal-Wallis tests to compare fruits per tree and fruit abundance between coarse grain categories. In order to assess differences in the degree of concordance across trophic levels, we first transformed our continuous density data to ordinal data. Ordination methods are described in detail in Appendix S2. The degree of concordance between ordinal densities for linked trophic pairs (tree-fly and fly-wasp) was quantified for the entire data set and for each coarse grain subset of sites using Kendall's tau-b correlations. We assessed differences between the resulting correlation coefficients, both within trophic levels across categories and within categories across trophic levels, via Fisher's z tests. If trophic levels are decoupled with intense landcover, we expected to see a significantly lower correlation between parasites and hosts in urban/suburban than in natural or agricultural landcover within a given trophic level. Further, if higher trophic levels have a greater degree of trophic decoupling with intense landcover, correlation with host densities is expected to be significantly lower in wasps than flies in the most intense (urban/suburban) landcover categories.

### Geospatial Analyses

We carried out spatially explicit analyses of organismal distributions to inform our understanding of spatial dynamics in the species under study. Ultimately, these analyses allowed us to assess the relative importance of landcover identity (i.e. level and type of land management/development) and scale (i.e. grain and extent) in resolving the degree of trophic decoupling observed between linked species. Average nearest neighbor analyses were carried out in ArcInfo to determine whether species under study exhibited clumped versus dispersed spatial distributions. This method calculates an index based on distances between sites where the species of interest is present and compares the observed distribution to the null hypothesis that sites are randomly distributed with respect to one another. We also performed local and global Moran's I statistics to detect positive and negative spatial autocorrelation (SA) for herbivory and parasitism rates. SA in species density may indicate processes such as conspecific attraction/population growth (positive SA) or dispersal limitation (negative SA) [35]. See Appendix S3 for a detailed description of SA analyses.

To assess potential impacts of soil characteristics on insect distributions (as a proxy for host plant quality differences), we compared soil attributes from the Soil Survey Geographical database [36] (water availability, phosphorous and potassium levels) with insect herbivory and parasitism via Spearman's ρ and Kruskal-Wallis tests. Water availability is likely important for host plant quality as both walnut and black cherry trees are mesic species. Soil concentrations of both phosphorous and potassium are a good measure of nutrient content in the host because levels of these nutrients in soils tend to be limiting for plants [37]. Phosphorous may be a particularly good proxy for host quality as this nutrient may limit larval growth rates [38] and rates of herbivory in general [39]. Conversely, an inverse relationship between potassium levels and soybean aphid (*Aphis glycines*) population growth rates was attributed to stress-induced increases in plant nitrogen content [40].

We performed linear spatial regression of herbivory and parasitism rates against fine grain landcover proportions at six spatial extents (i.e. buffer radii) to determine which landcover types best predict insect distributions at which spatial scale (in GeoDA [41]). To assess the hypothesis that more intense landcover would disrupt trophic interactions to a greater extent in urban versus agricultural land, we performed this analysis for the entire landscape as well as urban and agricultural subsets of sites. Natural areas represented an insufficient proportion of the study landscape to be included in this analysis (i.e. the natural subset of sites was too small to perform a regression analysis). GeoDA is coupled to ArcInfo such that large datasets (i.e. landcover proportions for nine categories at each of six spatial extents) are easily managed, queried, and analyzed. Wind- versus insect-pollinated crop landcover categories were treated as separate independent variables in these analyses because the two categories consistently yielded regression coefficients with opposite signs. Because GeoDA employs linear-based spatial regression modeling, we natural log-transformed all dependent variables (adding 0.025 to each value for cherry-associated insects to allow for transformation of zeros). The natural log transformation was used because walnut fly herbivory rates were >1 so that conventional arcsine square root or logit transformations were not possible. For cherry fly and wasp densities, the natural log transformation normalized the distributions equally well or better when compared to arcsine square root and logit transformations via Shapiro-Wilk tests of normality in SPSS. Spatial regression in GeoDA tests three different models, including ordinary least squares (OLS), spatial error, and spatial lag, to determine best fit. The spatial error model is appropriate when spatial dependence exists in the residuals due to underlying heterogeneous spatial structure or covariates not accounted for (i.e. spatial autocorrelation in the residuals), while a spatial lag model is appropriate when the value of a given dependent variable is influenced by neighboring values (i.e. spatial autocorrelation within a variable) [42]. Regression was carried out in a step-wise manner using landcover proportions around sites at each individual spatial extent respectively. The best fit for all models performed was ordinary least squares.

### Post-hoc Analyses: Multi-scale Generalized Linear Modeling

We performed several analyses in a generalized linear modeling framework in SPSS to assess the validity of results indicating potential importance of specific spatial scales (or sets of spatial scales) in explaining the observed insect distributions. This common framework facilitated comparisons of the relative strength and validity of associations detected both within separate aspects of scale (i.e. variation in grain at which landcover is categorized and variation in extent over which proportions are measured) and between these aspects of scale. First, to directly assess effects of landcover categorization across scales, we modeled main effects of both landcover grain categorizations as independent factors, as well as the interaction between fine and coarse grain categories, with insect densities respectively as response variables. Next, to directly assess the relative strengths of multi-scale (multi-buffer radius distance) associations identified between insect densities and landcover proportions, we modeled main effects on insect densities individually as response variables with landcover proportions in individual categories at disparate scales as independent covariates (including an interaction between spatial scales in the model as with grain above). We performed this analysis using densities of each of the three insect species in combination with landcover proportions at local (250 m) versus larger (3 and 4 km) buffer radii. These proportions included the four urban categories, their pooled totals (the total urban landcover proportion), open water, and wooded landcover.

## Results

### Summary Data

Table 2 summarizes all collection data for both systems, including global site occupancy data and numbers collected. Across the entire region, we identified general differences between the walnut and cherry systems in site occupancy and trophic interactions. Black walnut and black cherry host trees were present on 21.6% (N = 249) and 27.2% (N = 249) of the random study sites respectively (Table 2). Cultivated and volunteer trees of both species were found singly and in clusters in edge and open habitat types across the landscape, including yards, parks, roadside ditches, riparian strips, and forested edges. No sites with walnut trees lacked walnut fruits, though individual trees at some sites were occasionally bare of fruit. By contrast, cherry trees fruited on only 70% of sites with trees. We collected an average of 103.036 walnut fruits per site (±9.126 SE, range = 2-246, N = 54) and 434.167 cherry fruits per site (±89.129 SE, range = 3-3440, N = 42) from sites where cherry trees fruited. Walnut fruit collections yielded on average 1346.321 fly larvae per site (±242.920 SE, range = 9-6995), while an average of 20.810 fly larvae per site (±8.242, range = 0-305) emerged from cherry collections. Mean walnut fly herbivory rates were orders of magnitude higher than cherry fly herbivory (>10 versus <0.1 larvae per fruit, Table 2). Distributions of walnut fly herbivory rates differed significantly with levels of subsoil phosphorous so that more phosphorous was associated with lower herbivory (Kruskal-Wallis test: p = 0.026), but phosphorous levels did not vary with coarse landcover (Fisher exact test: p = 0.078). No other significant effects of soil attributes (a proxy for host quality) on fly herbivory rates were found in either species (Table S2).

**Table 2:** Summary statistics for host trees and insects sampled.

		WALNUT SYTEM			CHERRY SYSTEM		
		Trees	Flies	Wasps	Trees	Flies	Wasps
Entire landscape	Raw mean density	3.000±1.000	13.312±1.970	0.116±0.031	7.000±1.000	0.027±0.010	0.048±0.015
	Total sites occupied	54	54	10	68	24	10
	Total sites sampled	248	54	11	248	42	24
	Total collected	5770	75349/723	85	18235	874	78
N (Coarse grain)	Natural	24	6	2	24	4	4
	Agricultural	161	25	4	161	24	13
	Urban/Suburban	63	23	5	63	14	7
N (Fine grain)	Wooded	45	9	6	45	13	11
	Herbaceous	54	10	NA	54	11	8
	Cropland	91	10	3	91	8	3
	OD	26	9	2	26	6	3
	LDD	13	5	NA	13	3	0
	MDD	9	1	NA	9	1	0
	HDD	10	1	NA	10	0	0

Total raw mean densities ± SE are shown for each taxon. Tree densities are per site, fly densities are in larvae per fruit, cherry wasp densities are adults per fly larvae, and walnut wasp densities are adults per fly pupae. Total sites sampled includes all surveyed sites for trees (excluding one aquatic site), and for flies and wasps includes all sites where fruit and fly hosts were present respectively. Total collected for trees represents total fruits collected. Corresponding numbers for walnut flies show larvae from fruits/pupae from soil. NA indicates plot subsets not sampled.

### Site Occupancy

**Figure 2:**
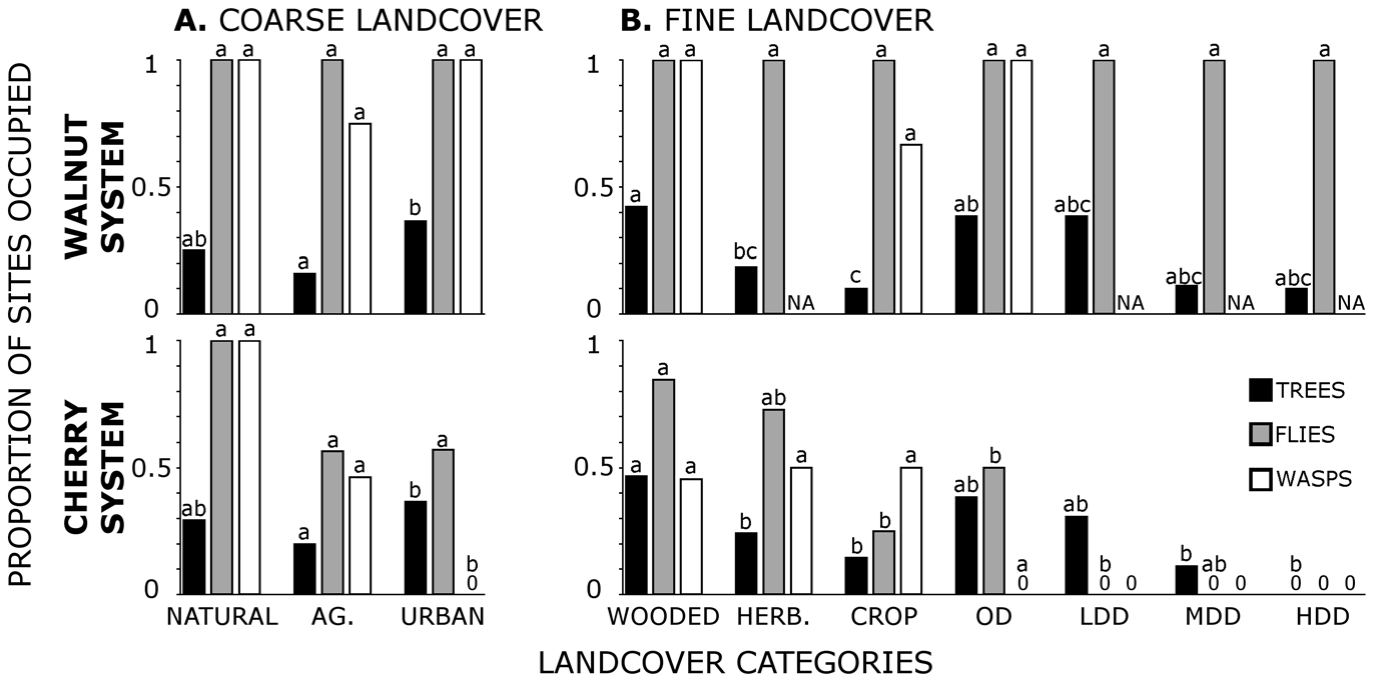
Occupancy of sites by trees, flies and parasitoid wasps at coarse and fine grain resolutions. Letters in common indicate no significant difference via pairwise Fisher's exact test (p<0.05) *within a trophic level*. P-values are shown in Table S3. Category names are explained in Table 1 and sample sizes are shown in Table 2. For trees, proportions are in relation to the total number of sites in each landcover class. Only sites with fruits were used to calculate proportions of sites with flies. Similarly, only sites with flies were used to calculate proportions of sites with wasps. **A.** Proportions of sites occupied across coarse grain landcover categories, **B.** Proportions of sites occupied across fine grain landcover categories.

Occupancy patterns in coarse and fine grain landcover were similar between walnut and cherry trees, but different between walnut- versus cherry-associated insects (Figure 2A & 2B, Table S3A & S3B). Walnut-associated flies and parasitoid wasps were present on almost all sites with walnut trees regardless of landcover, while site occupancy by cherry-associated insects varied with landcover type. Distributions of occupied sites in the cherry system showed increased sensitivity to fine grain landcover type with increasing trophic level (Figure 2B). Cherry flies were absent in the two most intense urban/suburban landcover categories in which host trees were present, while cherry wasps were absent in all urban/suburban categories.

Matrix analyses showed significant differences in occupancy outcomes among fine and coarse grain categories for both systems. Because walnut flies occupied 100% of sites with walnut trees (i.e. the only observed outcomes were presence of trees and flies or absence of both), this analysis detected significant differences among landcover categories but not among trophic levels (coarse grain: Χ^2^ = 11.863, df = 2, p = 0.003; fine grain: Χ^2^ = 13.159, df = 6, p = 0.041; Table S4A). This difference was driven at coarse grain resolution by excess walnut tree/fly presence (relative to expected counts) in urban areas with a corresponding deficit in agricultural areas. Similarly, fine grain differences in walnut tree/fly presence were driven by occupancy deficits in cropland and excesses relative to expectations in two urban categories (OD and LD). For the cherry system, significantly different occupancy patterns were found across trophic levels among landcover categories (coarse grain: Χ^2^ = 21.591, df = 6, p = 0.001; fine grain: Χ^2^ = 30.268, df = 6, p = 0.035; Table S4B). Significant coarse grain differences reflect a deficit of sites with both cherry trees and flies but no wasps in natural areas, with a corresponding excess of these sites in urban/suburban landcover. Further, natural sites showed an excess of complete tri-trophic occupancy, while urban sites had a deficit of this occupancy pattern. Significant fine grain differences show similar dynamics between wooded and open development categories (excess tri-trophic occupancies in wooded areas, and deficiencies in OD).

### Organismal Density

**Figure 3:**
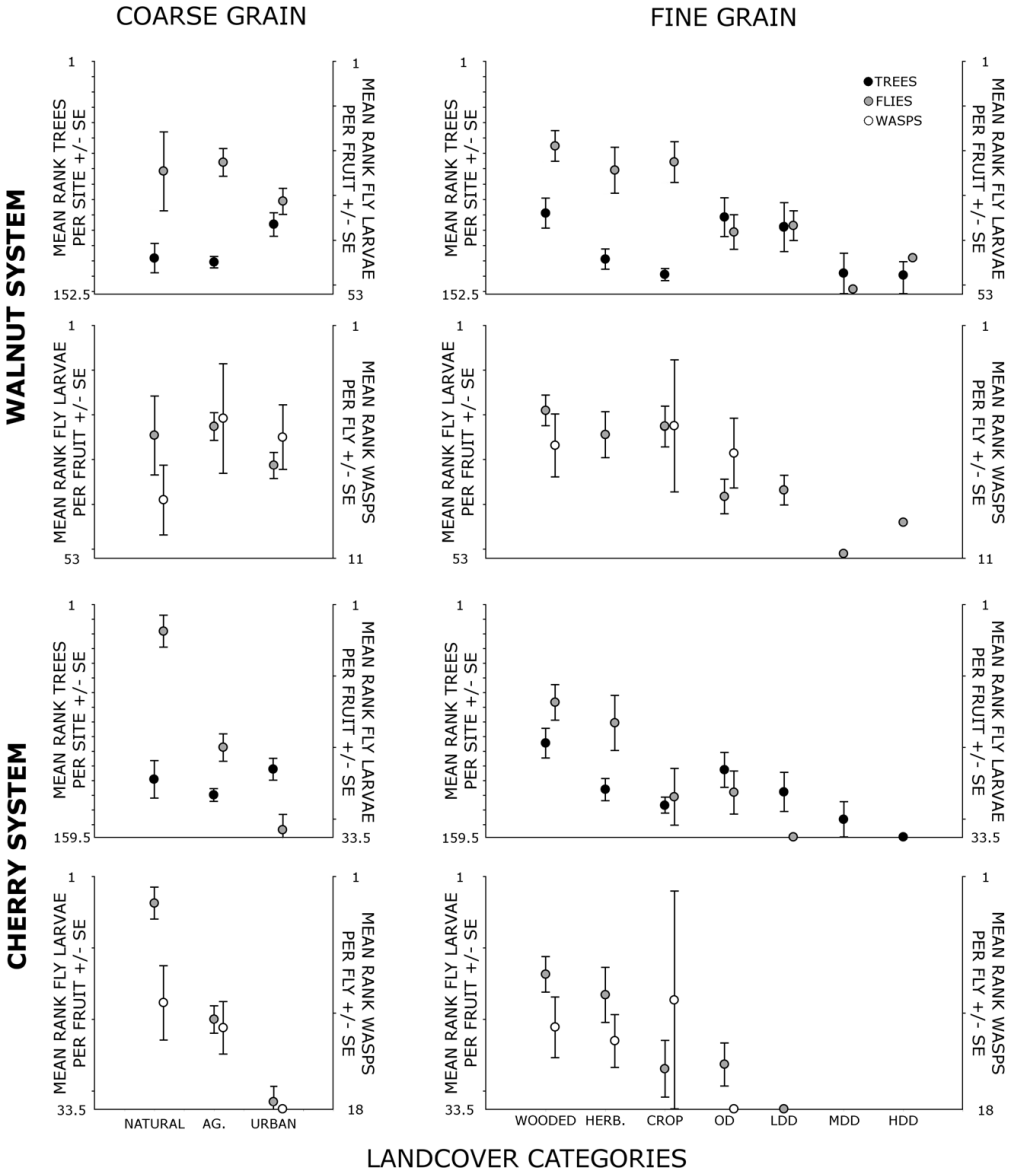
Mean rank tree density and insects parasitism rates (± SE) compared between trophic levels. Trees are symbolized with black circles, flies with gray circles, and wasps with white circles. Low mean ranks represent higher densities/parasitism rates and vice versa. Ranges of raw values corresponding to ranks are: walnut trees per site, 0-66; cherry trees per site, 0-180; walnut fly larvae per fruit, 0.250-75.500; cherry fly larvae per fruit, 0.000-0.401; walnut wasps per fly pupae, 0.000-0.312; cherry wasps per fly larvae, 0.000-0.212. P-values reflecting significance of differences across landcover within trophic levels are reported in Table S5.

Densities of host trees and flies also varied with coarse grain landcover for both cherry and walnut systems. Significantly higher mean rank host tree densities were found for both systems in urban/suburban versus agricultural landcover. Higher tree densities were not tracked by fly densities in the same categories for either system (Figure 3, Table S1A, Table S5A). Likewise, fine grain herbivory rates in both fly species failed to track host tree density in urban/suburban landcover types, with significantly lower larval density in built landcover categories compared to natural areas (Table S1B, Table S5B). For walnut-associated flies, urban/suburban densities were also lower than in cropland. Variation in parasitism rates tracked coarse grain but not fine grain infestation patterns of their host flies (Figure 3). At coarse grain, densities of both wasp species were lower in urban/suburban landcover than in agricultural landcover, while densities of walnut and cherry wasps did not differ across fine grain categories. We found no significant differences in fruits per tree (Kruskal-Wallis test, walnut: p = 0.328; cherry: p = 0.641) or mean fruit abundance (Kruskal-Wallis test, walnut: p = 0.102; cherry: p = 0.373) across coarse grain categories for either system.

Comparison of correlation coefficients with trophic level and coarse landcover showed mixed results between walnut and cherry systems. Correlations between walnut tree densities and fly densities, measured by Kendall's tau-b, were very high and did not differ significantly with landcover (Table 3). In the cherry system, by contrast, correlations between trophic levels (both tree-fly and fly-wasp) were significantly lower (via Fisher z test) in urban/suburban landcover than in either natural landcover (tree-fly: p = 0.039; fly-wasp: p = <0.001) or agricultural landcover (tree-fly: p = 0.039; fly-wasp: p = <0.001) (Table 3). Further, the correlation between cherry fly and tree density in urban/suburban landcover was significantly higher than between wasps and flies (p = 0.014). No other pairwise differences between correlation coefficients were significant.

**Table 3:** Inter-trophic correlations across coarse grain landcover categories.

	WALNUT TREE VERSUS FLY ORDINAL DENSITY			CHERRY TREE VERSUS FLY ORDINAL DENSITY			CHERRY FLY VERSUS WASP ORDINAL DENSITY		
	Correlation Coefficient	P	N	Correlation Coefficient	P	N	Correlation Coefficient	P	N
Entire landscape	0.952	<0.001	248	0.546	<0.001	248	0.627	<0.001	248
Natural	0.967^a^	<0.001	24	0.690^a^	0.001	24	0.871^a^	<0.001	24
Agricultural	0.965^a^	<0.001	161	0.585^a^	<0.001	161	0.685^a^	<0.001	161
Urban/Suburban	0.919^a^	<0.001	63	0.382^b^*	0.002	63	0.000^b^*	-	63

Kendall's tau-b correlation coefficients, p-values, and sample sizes are shown for comparisons of ordinal densities between linked trophic levels. Letters in common indicate no significant difference between correlation coefficients via Fisher's z compared across landcover categories (within a bi-trophic correlation comparison) at a threshold of p = 0.05. ‘*’ represents a significant pairwise difference in correlation coefficients between trophic levels (within a category and between two bi-trophic correlations in the cherry system).

### Geospatial Analyses

**Figure 4:**
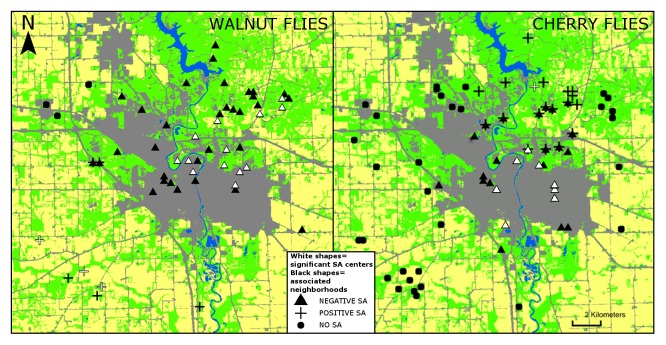
Local spatial autocorrelation (SA) in parasitism rates for cherry and walnut flies. Symbols indicate sites with positive (plus signs), negative (triangles), and no autocorrelation (dots). Symbols indicating centers of significant local autocorrelation (Moran's I, p<0.05) are shown in white, while all others are shown in black. Landscape areas presented include urban/suburban development (gray), agricultural land (yellow), “natural” wooded and herbaceous areas (green), and water (blue). More information regarding interpretation of this figure is provided in Appendix S3.

Average nearest neighbor analyses showed clustered distributions for both cherry trees (z = -1.89, p = 0.059) and cherry flies (z = -2.83, p<0.01), while walnut tree and walnut fly distributions did not differ from random expectations (z = −1.61, p = 0.11 [walnut flies were found wherever walnuts occurred]). Cherry wasps, by contrast, were significantly dispersed (z = 2.21, p = 0.027). Herbivory rates for both walnut and cherry flies show significant negative local SA centered within urban/suburban landcover in 69% (n = 13) of cases for walnut flies and all (n = 8) cases for cherry flies (Figure 4). Significant positive local SA in herbivory was found in agricultural development for walnut flies and natural areas for cherry flies. We did not detect global SA in fly herbivory rates for either system or any level of SA for cherry wasp parasitism rates. Sampling was not sufficient to assess SA for walnut wasps (because N<24; see [42]).

Linear regression models describing spatial variation in fly herbivory were most informative when coarse grain urban/suburban and agricultural landcover areas were considered separately (Table 4, Table S6). For walnut flies in agricultural landcover, 38.5% of variation in rates of herbivory was explained by proportions of all built landcover classes (HDD, MDD, LDD, and OD), wooded areas, and open water within a 2 km radius of site centers. Landcover proportions including insect-pollinated cropland, low density built development, wooded areas, and open water explained 14.9% of walnut herbivory variation across the entire landscape at a 3 km radius. Proportions of high and low density built development explained11.8% of walnut herbivory in urban/suburban landcover at a 500 m radius. Cherry fly herbivory rates were best explained in urban landcover, where proportions of insect- and wind-pollinated cropland, high and medium density built development, wooded areas, and open water at local (250 m) radii explained up to 81.3% of observed variation. Proportions of insect- and wind- pollinated cropland, high density built development, wooded areas, and water explained 29.2% (at 4 km) of variation in cherry fly rates of herbivory over the entire landscape. Proportions of insect-pollinated cropland, low density built development, and open water explained 39.4% (at 250 m) of variation in cherry fly herbivory rates in agricultural landcover. Landcover proportions including insect-pollinated cropland, high and medium density built development, wooded areas, and open water explained 54.3% (at 250 m) of variation in cherry wasp parasitism rates across the landscape. Limited site occupancy by cherry wasps precluded regression analyses within coarse grain categories, and sample sizes for walnut wasps were too small for spatial regression.

**Table 4:** Linear regression models in which landcover best predicted fly and wasp parasitism rates.

		Extent (radius)	Adj. R^2^	R^2^	P	F	N	Significant parameter(s)	Direction of effect
Walnut fly larvae per fruit	Entire landscape	3 km	0.149	0.213	0.017	3.322	54	LDD	negative
								Water	negative
	Agricultural	2 km	0.385	0.533	0.015	3.613	25	MDD	negative
								LDD	positive
								OD	negative
	Urban/Suburban	500 m	0.118	0.198	0.110	2.468	23	NA	NA
Cherry fly larvae per fruit	Entire landscape	4 km	0.292	0.380	0.004	4.295	42	Water	positive
	Agricultural	250 m	0.394	0.481	0.007	5.561	23	IPC	positive
								Water	negative
								LDD	negative
	Urban/Suburban	250 m	0.813	0.899	0.003	10.424	14	IPC	positive
								WPC	negative
								Wooded	positive
Cherry wasps per larvae	Entire landscape	250 m	0.543	0.647	0.002	6.229	24	IPC	positive
								Wooded	positive

The best regression model is shown for each species and geographical subset of sites, including the individually significant parameters (p<0.05) and the direction of their effect for each model. The top five regression models for each species and geographical subset are shown in Table S6, including coefficients and significance for individual independent variables included in each model. Adj  =  adjusted.

### Post-hoc Analyses: Multi-scale Generalized Linear Modeling

Multi-scale comparisons showed mixed results with respect to main effects of categories at different grains and landcover proportions at different distances. Results showed a significant main effect of fine grain landcover on walnut fly densities (p<0.001, Table S7A), with the four urban categories as individually significant (negative) parameters (p<0.001–0.053). We found no significant main effects of the specific landcover proportions assessed (including individual and pooled urban landcover categories, wooded landcover, and open water) across any set of scales on walnut fly densities. Coarse grain landcover was found to have a significant main effect on cherry fly density (p = 0.001, Table S7B) with a significant interaction between coarse and fine grain categories (p = 0.013). This interaction was driven by an overall positive effect of fine grain herbaceous landcover on cherry fly density, the slope of which declined significantly in agricultural and urban landcover (relative to natural landcover) at coarse grain. For cherry fly density, the only landcover proportion variable with main effects at any scale was open water, which showed a significant positive effect at 4 km (p = 0.045, Table S8A). Further analyses to assess the extent to which this effect was detectable within coarse grain urban/suburban versus agricultural subsets showed significant negative main effects of the proportion of water only at 250 m distances, but, in urban/suburban sites, we also detected a significant positive interaction between 250 m and 4 km scales (Table S8A). We detected no significant main effects of categorical landcover variables on cherry wasp densities, but found a significant negative effect of the pooled proportion of urban landcover at the 250 m radius (p = 0.010, Table S8B) with a significant negative interaction between 250 m and 4 km proportions (p = 0.003). No other variable showed significant main effects on cherry wasp density at any scale assessed.

## Discussion

We found support for our hypothesis that urban/suburban development decouples trophic interactions between plants, phytophagous insects, and parasitoid wasps. Although both host tree species (*J. nigra* and *P. serotina*) were more abundant at urban/suburban sites than in agricultural areas, densities of their associated fruit flies (*R. suavis* and *R. cingulata*) and parasitoid wasps (*C. pomonellae* and *D. ferrugineum*) were significantly lower in urban landcover. At fine grain, fly species had lower densities than expected on fruits in open development and low density built development sites. Walnut fly and cherry fly rates of herbivory also both showed significant negative local SA centered on urban/suburban areas. Further, densities for both species were best predicted by landcover variables at the most local (250–500 m) scales assessed. Taken together, these results indicate that fly distributions relative to host resources are limited in urban/suburban landcover compared with other landcover types. Coarse grain wasp parasitism rates followed patterns similar to herbivores (densities in urban sites were depressed relative to densities in agricultural sites) to the point that cherry wasps were entirely extirpated from urban/suburban areas. These results support our second hypothesis: landcover impacts parasitoid natural enemies to a greater degree than their herbivore hosts. Our direct comparisons of inter-trophic correlations across landcover categories provide additional support for both of our hypotheses. In the cherry system, correlations between inter-trophic densities were significantly lower in urban/suburban compared to natural and agricultural areas for both trophic levels, and, within urban/suburban cover, the fly-wasp correlation was significantly lower than the tree-fly correlation. By contrast, this analysis showed walnut fly densities to be highly concordant with those of their host trees across landcover types.

While all four insect species distributions showed sensitivity to urban/suburban land use, comparison of insect occupancy data between systems indicated disparate spatial population dynamics. Prevailing metapopulation models predict some proportion of habitat patches will be empty due to stochastic local extinctions. Site occupancy by flies associated with walnuts was complete (100%), and nearly so for walnut wasps (90.9%). This level of patch occupancy implicates a balanced dispersal model, wherein variation in local walnut-associated insect densities and other demographic traits on patches would be driven by differences in immigration rates between habitat patches that may be entirely occupied [43], [44]. While other metapopulation models view populations as limited by competition so that dispersal is predicted to be positively density-dependent, the balanced dispersal model predicts net immigration into higher density populations because these patches have higher carrying capacities [44], [45]. Complete site occupancy by walnut flies may also be a function of life history traits; unlike cherry flies, which lay a single egg per fruit, walnut flies oviposit clutches of up to 100 eggs [25]. This latter strategy is expected to result in larger local population sizes [46] and more dispersing individuals [47]. It may also provide a more stable resource base for parasitoids. Walnut wasps emerging under trees should not need to disperse long distances to locate hosts if their fly host resources are predictably spatially and temporally stable. In contrast to the apparent balanced dispersal in walnut flies, site occupancy by cherry flies was consistent with classic metapopulation models predicting spatially intermittent occupation of sites [45]. Differences in site occupancy by flies are explicable via these contrasting demographic regimes; balanced dispersal in a gregarious insect predicts substantially different patterns of site occupancy than predictions under other metapopulation assumptions. Demographic traits of each fly species also have important implications for their parasitoid wasps. Because traditional metapopulation dynamics predict that some host tree patches will be unoccupied by cherry flies, and because trophic levels in stacked specialist systems colonize patches sequentially [48], cherry-associated parasitoid wasps colonizing patches in a given landscape will be limited to the subset of patches with available fly hosts. Such bottom-up resource limitation may explain differences between wasp species in sensitivity to intense land use (i.e. bottom-up resource instability for the cherry system versus relative stability for the walnut system). Despite these system-specific differences, all insects still showed depressed densities and/or absences in similar urban/suburban landcover areas.

Potential proximate causes for the weakened or absent inter-trophic species associations in both systems include landcover-specific differences in host availability (quantity or quality), spatial configurations of hosts, and local dispersal limitation. Given that fruiting walnut and cherry trees were more abundant in urban areas, lower urban host quality may explain insect distributions better than sheer host numbers. Our finding that herbivory rates did not vary with most soil characteristics (as a proxy for host plant quality) argues against this explanation. The one exception was a significant effect of phosphorous on walnut fly densities. Higher soil phosphorous concentrations have been associated with higher rates of insect herbivory [39], but our results showed significantly lower herbivory rates with increased phosphorous and no association between this trend and landcover. Spatial configurations of hosts provide another alternative explanation for limited insect distributions; urban/suburban landcover is characterized by higher densities of habitat edges and edge to area ratios [49], which can depress biological diversity due to increased biotic and abiotic exposure to forces such as predation, competition, and microclimatic fluctuations [50], [51]. However, walnut and cherry trees are edge specialists [21], [22], so resources for sequential trophic levels (fruit and fly hosts respectively) are concentrated in edges in all landcover types. Positive edge effects on species diversity are therefore predicted [52]. More urban/suburban edges should result in more habitat space for these specialist insect species. This logic predicts higher insect densities in urban sites—a prediction not supported by our results.

Dispersal limitation currently provides the most robust explanation for patterns of spatial dispersion among species in our study. Our finding of negative local SA for both fly species in urban landcover supports this proximate explanation of the observed decoupling of trophic links in urban areas. While positive SA in the abundances and/or densities of populations may implicate any number of demographic processes (e.g. population growth or conspecific attraction), negative SA primarily indicates dispersal limitation [53]. Urban built structures may represent physical barriers for dispersing insects, and urban dispersal is often associated with increased mortality due to higher urban densities of insectivores such as birds [54]. Absence of cherry flies and wasps from sites in fine grain built categories with ample host resources is also consistent with urban-associated matrix impenetrability.

Differences in the spatial scales of insect responses to landcover provide some further support for dispersal limitation in urban landcover, but also suggest that different processes may be at work at different spatial scales [see 55]. Herbivory was best explained by surrounding landcover composition at 3–4 km radii across all sites combined for both fly species, while walnut flies responded to urban landcover at 500 m radii in urban/suburban landcover, and cherry flies responded at 250 m radii in both urban and agricultural areas. Cherry wasps, by contrast, responded at the 250 m extent across all sites combined. Because the scale at which a species responds to landcover may be indicative of dispersal distances, these local scale (250–500 m) responses to landcover are consistent with dispersal limitation in general for the cherry system, and specific to urban landcover for walnut flies [56]. Further, the very fine scale response in cherry wasps across all pooled sites is consistent with greater dispersal limitation at this higher trophic level. Substantial differences in the effective scale of herbivore responses to landcover when sites were pooled across the entire landscape versus in urban and/or agricultural subsets alone also indicate that limitations on dispersal differ mechanistically with differences in landcover. For example, very fine scale (250–500 m extent) responses in urban/suburban areas may be due to demographic or physical constraints (e.g. barriers) on distributions, while the larger scale (3–4 km extent) response that is resolved across the entire landscape sampled (all sites pooled) may be indicative of distance-based dispersal limitation.

Generalized linear modeling across scales in the cherry system supported results from regression analyses indicating responses at multiple distinct scales, although, in some cases insect responses to different scales were not independent. Coarse grain landcover had a significant main effect on cherry fly densities, with a significant interaction between coarse and fine grain categories. Specifically, this result showed a strong positive effect of site level herbaceous landcover on cherry fly density that depended on dominance of natural landcover at coarse grain (i.e. the intensity of the positive effect declined significantly in agricultural and urban/suburban subsets of sites). Similarly, the intensity of a local (250 m radius), negative effect of increasing urban landcover proportion on cherry wasp density became more strongly negative with increasing proportion of urban landcover at the 4 km radius. These interactions between effects at separate spatial scales indicate that, even when patterns of trophic decoupling are well resolved at a single scale, multi-scale analyses may be needed to adequately characterize responses of trophic connectivity to landcover. These findings are analogous to those of a recent study in which responses of Western corn rootworm (*Diabrotica virgifera*) densities to local (1 km) field corn cover were shown to depend on the regional (20 km) proportion of field corn cover, underscoring the potential complexity of interactions between insect distributions, landcover patterns, and spatial scale [57]. In the walnut system, because no single landcover proportion variable had a main effect on herbivore density, comparisons of individual landcover proportions across scales were not informative. However, our finding that fine grain landcover had a significant main effect on walnut fly density, specifically due to negative effects of the urban categories, indicates that local (site level) landcover strongly impacts urban/suburban walnut fly distributions in addition to the larger (2–3 km) distances that were strongly supported for agricultural and pooled populations by our regression results. Taken together, the results of our spatial regression and generalized linear modeling analyses both indicate that spatial variation in the level of decoupling between trophic levels across the study landscape depend on landcover as well as spatial scale. Our results provided complimentary evidence that processes operating at both local (site level) and larger (3–4 km) scale mediated effects of landcover on trophic connectivity for the specialist insects under study. Further, spatial regression results showed that the landcover parameters most important in explaining spatial variation in herbivore density differed both between species and with urban/suburban versus agricultural landcover category at coarse grain. These findings underscore the potential complexity in relationships between scale and landcover that may underlie trophic interactions in human-dominated landscapes; impacts of landcover and scale on trophic connectivity may be highly specific to landcover type.

Local availability of specific host plant habitats in urban landcover is not sufficient to support the full diversity of associated insect assemblages found at a regional scale. In particular, urban and suburban landcover are associated with markedly depressed densities and/or absences of insects associated with existing plant habitats, and this effect is exaggerated at higher trophic levels. Future work will compare the same tri-trophic distributions across multiple years to assess patterns of population persistence over time. Further, *R. cingulata* and *R. suavis* distributions conform to contrasting theoretical dispersal regimes, providing a compelling framework for future prediction and modeling of dispersal from a landscape genetic perspective.

## Supporting Information

Appendix S1 **Geoprocessing of Landcover Data.**
(DOCX)

Appendix S2 **Ordination of Tri-trophic Density Data.**
(DOCX)

Appendix S3 **Local Spatial Autocorrelation Methods and Interpretation.**
(DOCX)

Figure S1 **The proportion of each fine grain landcover category composing each coarse grain landcover category.** Abbreviations used for fine grain landcover categories are shown in Table 1.
(JPG)

Table S1 **Coarse and fine grain mean rank tree densities and insect herbivory/parasitism rates ± standard error.** Raw mean densities/rates ± standard error are in parentheses. Higher ranks are indicative of lower density and vice versa. Full ranges of raw and rank values are shown in Figure 3. P-values are shown in Table S5. **A. Coarse grain.** Different letters indicate significantly different distributions via pair-wise Mann-Whitney U-tests (p<0.05) and refer to intra-specific comparisons within trophic levels (columns). **B. Fine grain.** Distributions for trees and flies (but not wasps) in both systems differed significantly across categories via Kruskal-Wallis tests (p<0.05). ‘NA’ indicates plot subsets not sampled and ‘-‘ indicates lack of host.
(DOCX)

Table S2 **Soil impacts on fly density.** Results are shown for Spearman's rho and Kruskal-Wallis comparisons of fly parasitism rates with USDA-NRCS Soil Survey Geological data as proxy for plant quality. Test statistics for correlations are shown in parenthesis.
(DOCX)

Table S3 **P-values for pair-wise Fisher**'**s exact tests comparing intraspecific site occupancy across coarse and fine grain landcover categories.** **A.** P-values associated with coarse grain site occupancy within each species. **B.** P-values associated with fine grain site occupancy within each species. * Indicates Bonferroni-corrected significance.
(DOCX)

Table S4 **Observed and expected occupancy outcomes in the walnut and cherry systems.** A. Observed and expected counts for all occupancy outcomes across trophic levels and landcover categories at fine and coarse grain in the walnut system. Each two digit code represents the two lower trophic levels (tree-fly). Presence  = 1 and absence  = 0. B. Observed and expected counts for all occupancy outcomes across trophic levels and landcover categories at fine and coarse grain in the cherry system. Each three digit code represents the three trophic levels (tree-fly-wasp). Presence  = 1 and absence  = 0.
(DOCX)

Table S5 **P-values for intraspecific density comparisons across coarse and fine grain landcover categories. A.** P-values associated with pair-wise Mann-Whitney U-tests comparing tree density, fly herbivory, and wasp parasitism rates across coarse grain landcover categories. **B.** P-values associated with Kruskal-Wallis tests comparing tree density, fly herbivory, and wasp parasitism rates across fine grain landcover categories.
(DOCX)

Table S6 **Top five regression models in which landcover proportions predicted herbivory and parasitism rates.** Results are shown at the level of the entire landscape and among urban/suburban and agricultural subsets of sites. Summary statistics for individual models are shown. Each model is designated with a number corresponding to the set of coefficients and p-values associated with individual independent variables within the model as shown in the lower half of the table. Statistics are not shown for urban/suburban models associated with walnut flies (*R. suavis*) because none of these models were significant. EL =  entire landscape, AG =  agricultural site subset, URB =  urban/suburban site subset.
(DOCX)

Table S7 **Significant main effects of landcover categories on herbivore densities.** Results are shown for significant main effects found in generalized linear models designed to assess responses of insect densities to fine versus coarse landcover categories, and potential interactions between the two grains. Direction of effect is indicated for significant parameters. A. Main effects of landcover category on walnut fly density. B. Main effects of landcover category on cherry fly density.
(DOCX)

Table S8 **Significant main effects of landcover proportions on densities in cherry-associated insects.** Results are shown for significant main effects found in generalized linear models designed to assess responses of insect densities to local (250 m) versus larger (4 km) proportions of individual landcover variables, and potential interactions between the two buffer distances. Direction of effect is indicated for significant main effects. A. Main effects of the proportion of open water on cherry fly density. Results for models within agricultural and urban/suburban subsets of sites are also shown. B. Main effects of the proportion of total pooled urban landcover on cherry wasp density.
(DOCX)
