# Supplementary material for: Urban Land Use Decouples Plant-Herbivore-Parasitoid Interactions at Multiple Spatial Scales
Source: PLoS One. 2014 Jul 14;9(7):e102127. doi: 10.1371/journal.pone.0102127 (PMC4096920; doi:10.1371/journal.pone.0102127)
Supplement: Appendix S1 — Geoprocessing of Landcover Data. (DOCX) [file pone.0102127.s001.docx]

**Appendix S1. Geoprocessing of Landcover Data**

We performed our geoprocessing by first converting site polygons to point features at the centroids of sites and generating a buffer layer for each radius. We then clipped the CropScape data layer (which had been converted to a vector shapefile in ArcInfo) using the buffer layer, and performed an intersect operation so that land cover polygon boundaries in overlapping buffers conformed to site-associated buffer boundaries. Next we calculated the area (m^2^) of each land cover polygon. In order to capture one-to-many relationships between land cover polygons and buffers, we performed a spatial join operation between the two data layers.
