# Supplementary material for: Urban Land Use Decouples Plant-Herbivore-Parasitoid Interactions at Multiple Spatial Scales
Source: PLoS One. 2014 Jul 14;9(7):e102127. doi: 10.1371/journal.pone.0102127 (PMC4096920; doi:10.1371/journal.pone.0102127)
Supplement: Appendix S2 — Ordination of Tri-trophic Density Data. (DOCX) [file pone.0102127.s002.docx]

**Appendix S2.** **Ordination of Tri-trophic Density Data**

Tree, fly, and wasp densities were divided into bins for ordination using several different methods, each progressively subdividing the observed values more finely. In all methods, absences were coded as “0”, otherwise, methods were specified as follows:

**Walnut system**, Method 1: tree bins= 1- 10, >10 trees per site; fly bins= 1- 10, >10 larvae per fruit. Method 2: tree bins= 1- 10, >10- 100, >100 trees per site; fly bins= <5, >5- 10, >10 larvae per fruit. Method 3: tree bins= 1- 10, >10- 50, >50- 100, >100 trees per site; fly bins= <5, >5- 10, >10- 20, >20 larvae per fruit.

**Cherry system**, Method 1: tree bins= 1-20, >20 trees per site; fly bins= <0.05, >0.05 larvae per fruit; wasp bins= <0.10, >0.10 wasps per larvae. Method 2: tree bins= 1- 20, >20- 100, >100 trees per site; fly bins= < 0.05, 0.05- 0.10, >0.10 larvae per fruit; wasp bins= < 0.05, 0.05- 0.10, >0.10 wasps per larvae. Method 3: tree bins= 1- 3, >3- 20, >20- 100, >100 trees per site; (no additional subdivision for flies or wasps was practical).

Kendall’s tau-b correlations between linked trophic levels were performed between all pairwise ordination methods to ensure that the method of subdivision into bins did not impact the results. Correlation results followed identical trends across systems and landcover categories regardless of ordination methods. All results reported in the main text correspond to the first ordination method described for both the walnut and cherry system (“Method 1”) as this method was the most conservative for displaying Fisher z transformation results (i.e. yielded the smallest differences in correlation between landscape categories).
