# Supplementary material for: Urban Land Use Decouples Plant-Herbivore-Parasitoid Interactions at Multiple Spatial Scales
Source: PLoS One. 2014 Jul 14;9(7):e102127. doi: 10.1371/journal.pone.0102127 (PMC4096920; doi:10.1371/journal.pone.0102127)
Supplement: Appendix S3 — Local Spatial Autocorrelation Methods and Interpretation. (DOCX) [file pone.0102127.s003.docx]

**Appendix S3. Local Spatial Autocorrelation Methods and Interpretation**

We used GeoDA software to perform analyses designed to detect patterns of global (across all sites) and local (among subsets of sites) spatial autocorrelation (SA). While no global patterns of SA were detected, local SA was detected for both herbivore species. The map shown in Figure 4 depicts patterns of positive and negative local SA in the two herbivore species. The results generated from SA analyses performed in GeoDA included p-values for individual sites that were found to be low (or high) values surrounded by significantly more low (or high) herbivory sites than expected at random (compared to 5000 permutations of the data). The white symbols on the map are the sites with statistical significance at the p< 0.01 level, while black symbols indicate the neighbors associated with these sites. Thus, a site with both black symbols (i.e. both a cross and a triangle) was included in at least two neighborhoods (both for one site with positive SA and one site with negative SA). Conventionally, the entire neighborhood associated with a significant p-value is considered to be part of a cluster of local SA.
