# Supplementary figures and images for: Urban Land Use Decouples Plant-Herbivore-Parasitoid Interactions at Multiple Spatial Scales
Source: PLoS One. 2014 Jul 14;9(7):e102127. doi: 10.1371/journal.pone.0102127 (PMC4096920; doi:10.1371/journal.pone.0102127)

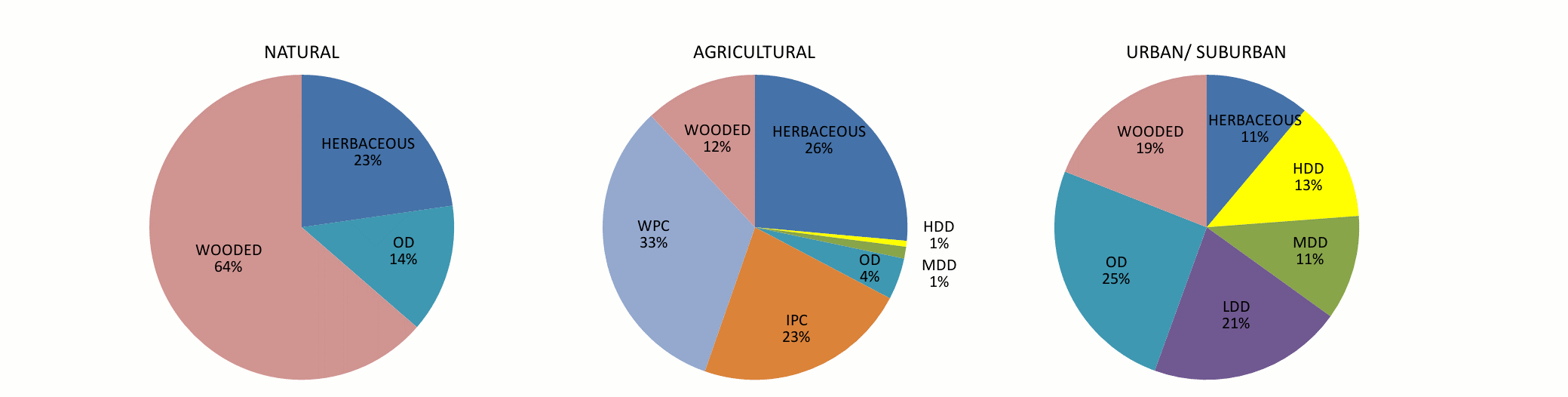

Supplement: Figure S1 — The proportion of each fine grain landcover category composing each coarse grain landcover category. Abbreviations used for fine grain landcover categories are shown in Table 1. (JPG) [file pone.0102127.s004.jpg]
