# Supplementary material for: Urban Land Use Decouples Plant-Herbivore-Parasitoid Interactions at Multiple Spatial Scales
Source: PLoS One. 2014 Jul 14;9(7):e102127. doi: 10.1371/journal.pone.0102127 (PMC4096920; doi:10.1371/journal.pone.0102127)
Supplement: Table S1 — Coarse and fine grain mean rank tree densities and insect herbivory/parasitism rates ± standard error. Raw mean densities/rates ± standard error are in parentheses. Higher ranks are indicative of lower density and vice versa. Full ranges of raw and rank values are shown in Figure 3. P-values are shown in Table S5. A. Coarse grain. Different letters indicate significantly different distributions via pair-wise Mann-Whitney U-tests (p<0.05) and refer to intra-specific comparisons within trophic levels (columns). B. Fine grain. Distributions for trees and flies (but not wasps) in both systems differed significantly across categories via Kruskal-Wallis tests (p<0.05). ‘NA’ indicates plot subsets not sampled and ‘-‘ indicates lack of host. (DOCX) [file pone.0102127.s005.docx]

| **Table S1.**  A. COARSE GRAIN | | | | **MEAN RANK ± SE (RAW MEAN ± SE)** | | | | |  |
| --- | --- | --- | --- | --- | --- | --- | --- | --- | --- |
|  |  | | | **TREES PER SITE** | | **FLIES PER FRUIT** | **WASPS PER FLY** | |  |
| **Walnut System** | Natural | | | 129.229 ± 9.543^ab^ (1.000 ± 0.866) | | 25.600 ± 8.824^ab^ (14.308 ± 5.009) | 8.500 ± 1.500  (0.034 ± 0.023) | |  |
|  | Agricultural | | | 131.861 ± 3.751^a^ (3.000 ± 0.704) | | 23.615 ± 3.116^a^ (16.387 ± 2.850) | 5.000 ± 2.357  (0.155 ± 0.055) | |  |
|  | Urban/ suburban | | | 107.294 ± 7.654^b^ (5.000 ± 1.383) | | 32.304 ± 2.917^b^ (10.393 ± 3.218) | 5.800 ± 1.390  (0.118 ± 0.050) | |  |
|  |  | | |  | |  |  | |  |
| **Cherry System** | Natural | | | 120.146 ± 12.826^ab^ (7.000 ± 2.690) | | 4.750 ± 2.250^a^  (0.147 ± 0.086) | 10.250 ± 2.720^a^  (0.080 ± 0.041) | |  |
|  | Agricultural | | | 130.864 ± 4.277^a^ (7.000 ± 1.782) | | 21.000 ± 2.490^b^  (0.019 ± 0.004) | 12.077 ± 1.913^ab^  (0.057 ± 0.021) | |  |
|  | Urban/ suburban | | | 113.206 ± 7.430^b^ (8.000 ± 2.892) | | 32.733 ± 2.125^b^  (0.006 ± 0.003) | 18.000 ± 0.000^b^  (0.000) | |  |
| B. FINE GRAIN | |  | **MEAN RANK ± SE (RAW MEAN ± SE)** | | | | | | |
|  | |  | **TREES PER SITE** | | **FLIES PER FRUIT** | | | **WASPS PER FLY** | |
| **Walnut System** | | Wooded | 100.044 ± 9.762  (7.000 ± 1.778) | | 20.000 ± 3.428  (20.941 ± 6.434) | | | 6.167 ± 1.352  (0.112 ± 0.050) | |
|  | | Herbaceous | 130.028 ± 6.581  (3.000 ± 1.501) | | 25.400 ± 5.132  (13.909 ± 3.657) | | | NA | |
|  | | Cropland | 140.102 ± 3.967  (1.000 ± 0.415) | | 23.556 ± 4.556  (14.280 ± 3.323) | | | 5.333 ± 2.848  (0.154 ± 0.077) | |
|  | | OD | 102.712 ± 12.687  (7.000 ± 2.261) | | 39.222 ± 3.890  (4.663 ± 1.406) | | | 6.500 ± 1.500  (0.070 ± 0.096) | |
|  | | LDD | 109.231 ± 15.943  (3.000 ± 1.481) | | 37.800 ± 3.292  (5.310 ± 1.237) | | | NA | |
|  | | MDD | 139.278 ± 13.222  (1.000 ± 0.889) | | 52.000 ± 0.000  (0.391 ± 0.000) | | | NA | |
|  | | HDD | 140.389 ± 12.111 | | 45.000 ± 0.000 | | | NA | |
|  | |  | (1.000 ± 0.559) | | (2.250 ± 0.000) | | |  | |
| **Cherry System** | | Wooded | 95.222 ± 10.094  (16.000 ± 4.495) | | 14.615 ± 2.501  (0.025 ± 0.004) | | | 12.000 ± 2.228  (0.065 ± 0.026) | |
|  | | Herbaceous | 126.963 ± 7.643  (5.000 ± 1.531) | | 17.500 ± 3.882  (0.061 ± 0.036) | | | 13.000 ± 1.936  (0.029 ± 0.013) | |
|  | | Cropland | 137.750 ± 5.222  (5.733 ± 2.551) | | 27.875 ± 3.981  (0.010 ± 0.008) | | | 10.000 ± 8.000  (0.100 ± 0.100) | |
|  | | OD | 113.500 ± 11.900  (9.000 ± 5.001) | | 27.250 ± 3.013  (0.004 ± 0.003) | | | 18.000 ± 0.000  (0.000) | |
|  | | LDD | 128.615 ± 13.379  (1.000 ± 0.308) | | 33.500 ± 0.000  (0.000) | | | - | |
|  | | MDD | 147.389 ± 12.111  (0.444 ± 0.444) | | - | | | - | |
|  | | HDD | 159.500 ± 0.000  (0.000) | | - | | | - | |

**Table S1. Coarse and fine grain mean rank tree densities and insect herbivory/ parasitism rates ± standard error.** Raw mean densities/ rates ± standard error are in parentheses. Higher ranks are indicative of lower density and vice versa. Full ranges of raw and rank values are shown in Figure 3. P-values are shown in Table S5. **A. Coarse grain.** Different letters indicate significantly different distributions via pair-wise Mann-Whitney U-tests (p< 0.05) and refer to intra-specific comparisons within trophic levels (columns). **B. Fine grain.** Distributions for trees and flies (but not wasps) in both systems differed significantly across categories via Kruskal-Wallis tests (p< 0.05). ‘NA’ indicates plot subsets not sampled and ‘-‘ indicates lack of host.
