# Supplementary material for: Urban Land Use Decouples Plant-Herbivore-Parasitoid Interactions at Multiple Spatial Scales
Source: PLoS One. 2014 Jul 14;9(7):e102127. doi: 10.1371/journal.pone.0102127 (PMC4096920; doi:10.1371/journal.pone.0102127)
Supplement: Table S2 — Soil impacts on fly density. Results are shown for Spearman's rho and Kruskal-Wallis comparisons of fly parasitism rates with USDA-NRCS Soil Survey Geological data as proxy for plant quality. Test statistics for correlations are shown in parenthesis. (DOCX) [file pone.0102127.s006.docx]

**Table S2.**

|  | **Soil variable** | **Test** | **P- value** |  |
| --- | --- | --- | --- | --- |
| ***R. suavis*** | average water capacity | Spearman's rho | 0.315 (-0.139) |  |
| *.* | subsoil phosphorous | Kruskal-Wallis | 0.026 |  |
|  | subsoil potassium | Kruskal-Wallis | 0.115 |  |
|  |  |  |  |  |
| ***R. cingulata*** | average water capacity | Spearman's rho | 0.964 (-0.007) |  |
|  | subsoil phosphorous | Kruskal-Wallis | 0.782 |  |
|  | subsoil potassium | Kruskal-Wallis | 0.170 |  |

**Table S2. Soil impacts on fly density.** Results are shown for Spearman’s rho and Kruskal-Wallis comparisons of fly parasitism rates with USDA-NRCS Soil Survey Geological data as proxy for plant quality. Test statistics for correlations are shown in parenthesis.
