# Supplementary material for: Urban Land Use Decouples Plant-Herbivore-Parasitoid Interactions at Multiple Spatial Scales
Source: PLoS One. 2014 Jul 14;9(7):e102127. doi: 10.1371/journal.pone.0102127 (PMC4096920; doi:10.1371/journal.pone.0102127)
Supplement: Table S3 — P-values for pair-wise Fisher's exact tests comparing intraspecific site occupancy across coarse and fine grain landcover categories. A. P-values associated with coarse grain site occupancy within each species. B. P-values associated with fine grain site occupancy within each species. * Indicates Bonferroni-corrected significance. (DOCX) [file pone.0102127.s007.docx]

**Table S3.**

| *J. nigra* |  |  |  | *P. serotina* | |  |
| --- | --- | --- | --- | --- | --- | --- |
|  | **AG** | **URBAN** |  |  | **AG** | **URBAN** |
| **NATURAL** | 0.382 | 0.446 |  | **NATURAL** | 0.605 | 0.459 |
| **AG** |  | 0.001 |  | **AG** |  | 0.012 |
|  |  |  |  |  |  |  |
| *R. suavis* |  |  |  | *R. cingulata* | |  |
|  | **AG** | **URBAN** |  |  | **AG** | **URBAN** |
| **NATURAL** | 1 | 1 |  | **NATURAL** | 0.264 | 0.245 |
| **AG** |  | 1 |  | **AG** |  | 1 |
|  |  |  |  |  |  |  |
| *C. pomonellae* | |  |  | *D. ferrugineum* | |  |
|  | **AG** | **URBAN** |  |  | **AG** | **URBAN** |
| **NATURAL** | 1 | 1 |  | **NATURAL** | 0.103 | 0.003 |
| **AG** |  | .444 |  | **AG** |  | 0.044 |

**A. COARSE GRAIN**

| *J. nigra* | (Bonferroni corrected α= 0.002) | | | | | |  | *P. serotina* | (Bonferroni corrected α= 0.002) | | | | | |
| --- | --- | --- | --- | --- | --- | --- | --- | --- | --- | --- | --- | --- | --- | --- |
|  | **HERB.** | **CROP** | **OD** | **LDD** | **MDD** | **HDD** |  |  | **HERB.** | **CROP** | **OD** | **LDD** | **MDD** | **HDD** |
| **WOODED** | 0.014 | >0.001* | 0.806 | 1 | 0.131 | 0.075 |  | **WOODED** | 0.013 | >0.001* | 0.324 | 0.212 | 0.031 | 0.006 |
| **HERB.** |  | 0.202 | 0.096 | 0.146 | 1 | 0.678 |  | **HERB.** |  | 0.402 | 0.301 | 0.736 | 0.437 | 0.101 |
| **CROP** |  |  | 0.001* | 0.015 | 1 | 1 |  | **CROP** |  |  | 0.062 | 0.458 | 0.694 | 0.205 |
| **OD** |  |  |  | 1 | 0.217 | 0.127 |  | **OD** |  |  |  | 0.733 | 0.217 | 0.034 |
| **LDD** |  |  |  |  | 0.333 | 0.179 |  | **LDD** |  |  |  |  | 0.36 | 0.104 |
| **MDD** |  |  |  |  |  | 1 |  | **MDD** |  |  |  |  |  | 0.474 |
|  |  |  |  |  |  |  |  |  |  |  |  |  |  |  |
|  |  |  |  |  |  |  |  |  |  |  |  |  |  |  |
| *R. suavis* |  | | | | |  |  | *R. cingulata* | (Bonferroni corrected α= 0.003) | | | | |  |
|  | **HERB.** | **CROP** | **OD** | **LDD** | **MDD** |  |  |  | **HERB.** | **CROP** | **OD** | **LDD** | **MDD** |  |
| **WOODED** | 1 | 1 | 1 | 1 | 1 |  |  | **WOODED** | 0.630 | 0.017 | 0.018 | 0.018 | 0.214 |  |
| **HERB.** |  | 1 | 1 | 1 | 1 |  |  | **HERB.** |  | 0.070 | 0.600 | 0.055 | 0.333 |  |
| **CROP** |  |  | 1 | 1 | 1 |  |  | **CROP** |  |  | 0.580 | 1 | 1 |  |
| **OD** |  |  |  | 1 | 1 |  |  | **OD** |  |  |  | 0.464 | 1 |  |
| **LDD** |  |  |  |  | 1 |  |  | **LDD** |  |  |  |  | 1 |  |
|  |  |  |  |  |  |  |  |  |  |  |  |  |  |  |
|  |  |  |  |  |  |  |  |  |  |  |  |  |  |  |
| *C. pomonellae* | | |  | |  | |  | *D. ferrugineum* | |  |  |  |  |  |
|  | **CROP** | **OD** | |  | |  |  |  | **HERB.** | **CROP** | **OD** |  |  |  |
| **WOODED** | 0.333 | 1 | |  | |  |  | **WOODED** | 1 | 1 | 0.258 |  |  |  |
| **CROP** |  | 1 | |  | |  |  | **HERB.** |  | 1 | 0.236 |  |  |  |
|  |  |  |  |  |  |  |  | **CROP** |  |  | 0.400 |  |  |  |

**B.** FINE GRAIN

**Table S3. P-values for pair-wise Fisher’s exact tests comparing intraspecific site occupancy across coarse and fine grain landcover categories.** **A.** P-values associated with coarse grain site occupancy within each species. **B.** P-values associated with fine grain site occupancy within each species. * Indicates Bonferroni-corrected significance.
