# Supplementary material for: Urban Land Use Decouples Plant-Herbivore-Parasitoid Interactions at Multiple Spatial Scales
Source: PLoS One. 2014 Jul 14;9(7):e102127. doi: 10.1371/journal.pone.0102127 (PMC4096920; doi:10.1371/journal.pone.0102127)
Supplement: Table S4 — Observed and expected occupancy outcomes in the walnut and cherry systems. A. Observed and expected counts for all occupancy outcomes across trophic levels and landcover categories at fine and coarse grain in the walnut system. Each two digit code represents the two lower trophic levels (tree-fly). Presence = 1 and absence = 0. B. Observed and expected counts for all occupancy outcomes across trophic levels and landcover categories at fine and coarse grain in the cherry system. Each three digit code represents the three trophic levels (tree-fly-wasp). Presence = 1 and absence = 0. (DOCX) [file pone.0102127.s008.docx]

**Table S4.**

A. WALNUT SYSTEM

| **Grain** |  | **Walnut Tree-Fly Occupancy Counts** | | | |
| --- | --- | --- | --- | --- | --- |
|  |  | **0-0** | | **1-1** | |
|  |  | **Observed** | **Expected** | **Observed** | **Expected** |
| **Coarse** | **Natural** | 18 | 18.774 | 6 | 5.226 |
|  | **Agricultural** | 136 | 125.944 | 25 | 35.056 |
|  | **Urban/ Suburban** | 40 | 49.282 | 23 | 13.718 |
|  |  |  |  |  |  |
| **Fine** | **Wooded** | 45 | 42.241 | 9 | 0.647 |
|  | **Herbaceous** | 54 | 50.065 | 10 | 1.111 |
|  | **Cropland** | 91 | 79.008 | 10 | 6.539 |
|  | **OD** | 26 | 27.379 | 9 | 0.250 |
|  | **LDD** | 13 | 14.081 | 5 | 0.298 |
|  | **MDD** | 9 | 7.823 | 1 | 0.636 |
|  | **HDD** | 10 | 8.605 | 1 | 0.813 |

B. CHERRY SYSTEM

| **Grain** |  | **0-0-0** | | **1-0-0** | | **1-1-0** | | **1-1-1** | |
| --- | --- | --- | --- | --- | --- | --- | --- | --- | --- |
|  |  | **Observed** | **Expected** | **Observed** | **Expected** | **Observed** | **Expected** | **Observed** | **Expected** |
| **Coarse** | **Natural** | 20 | 20.032 | 0 | 1.742 | 0 | 1.258 | 4 | 0.968 |
|  | **Agricultural** | 138 | 134.383 | 11 | 11.685 | 6 | 8.440 | 6 | 6.492 |
|  | **Urban/ Suburban** | 49 | 52.585 | 7 | 4.573 | 7 | 3.302 | 0 | 2.540 |
|  |  |  |  |  |  |  |  |  |  |
| **Fine** | **Wooded** | 32 | 37.379 | 2 | 3.085 | 6 | 2.722 | 5 | 1.815 |
|  | **Herbaceous** | 44 | 44.855 | 3 | 3.702 | 5 | 3.266 | 3 | 2.177 |
|  | **Cropland** | 83 | 75.589 | 5 | 6.237 | 1 | 5.504 | 2 | 3.670 |
|  | **OD** | 20 | 21.597 | 3 | 1.780 | 3 | 1.573 | 0 | 1.048 |
|  | **LDD** | 10 | 10.798 | 3 | 0.891 | 0 | 0.786 | 0 | 0.524 |
|  | **MDD** | 8 | 7.476 | 1 | 0.617 | 0 | 0.544 | 0 | 0.363 |
|  | **HDD** | 10 | 8.306 | 0 | 0.524 | 0 | 0.605 | 0 | 0.403 |

**Table S4. Observed and expected occupancy outcomes in the walnut and cherry systems.** A. Observed and expected counts for all occupancy outcomes across trophic levels and landcover categories at fine and coarse grain in the walnut system. Each two digit code represents the two lower trophic levels (tree-fly). Presence= 1 and absence= 0. B. Observed and expected counts for all occupancy outcomes across trophic levels and landcover categories at fine and coarse grain in the cherry system. Each three digit code represents the three trophic levels (tree-fly-wasp). Presence= 1 and absence= 0.
