# Supplementary material for: Urban Land Use Decouples Plant-Herbivore-Parasitoid Interactions at Multiple Spatial Scales
Source: PLoS One. 2014 Jul 14;9(7):e102127. doi: 10.1371/journal.pone.0102127 (PMC4096920; doi:10.1371/journal.pone.0102127)
Supplement: Table S5 — P-values for intraspecific density comparisons across coarse and fine grain landcover categories. A. P-values associated with pair-wise Mann-Whitney U-tests comparing tree density, fly herbivory, and wasp parasitism rates across coarse grain landcover categories. B. P-values associated with Kruskal-Wallis tests comparing tree density, fly herbivory, and wasp parasitism rates across fine grain landcover categories. (DOCX) [file pone.0102127.s009.docx]

**Table S5.**

| A. |  |  |  |  |  |  |
| --- | --- | --- | --- | --- | --- | --- |
| *J. nigra* |  |  |  | *P. serotina* |  |  |
|  | **AG** | **URBAN** |  |  | **AG** | **URBAN** |
| **NATURAL** | 0.733 | 0.093 |  | **NATURAL** | 0.383 | 0.713 |
| **AG** |  | 0.002 |  | **AG** |  | 0.034 |
|  |  |  |  |  |  |  |
| *R. suavis* |  |  |  | *R. cingulata* |  |  |
|  | **AG** | **URBAN** |  |  | **AG** | **URBAN** |
| **NATURAL** | 0.214 | 0.684 |  | **NATURAL** | 0.006 | 0.001 |
| **AG** |  | 0.050 |  | **AG** |  | 0.145 |
|  |  |  |  |  |  |  |
| *C. pomonellae* |  |  |  | *D. ferrugineum* |  |  |
|  | **AG** | **URBAN** |  |  | **AG** | **URBAN** |
| **NATURAL** | 0.533 | 0.381 |  | **NATURAL** | 0.202 | 0.010 |
| **AG** |  | 0.730 |  | **AG** |  | 0.172 |

| B. | **P-value** |  |  | **P-value** |
| --- | --- | --- | --- | --- |
| *J. nigra* | 0.002 |  | *P. serotina* | 0.008 |
| *R. suavis* | 0.023 |  | *R. cingulata* | 0.017 |
| *C. pomonellae* | 0.913 |  | *D. ferrugineum* | 0.465 |

**Table S5.** **P-values for intraspecific density comparisons across coarse and fine grain landcover categories. A.** P-values associated with pair-wise Mann-Whitney U-tests comparing tree density, fly herbivory, and wasp parasitism rates across coarse grain landcover categories. **B.** P-values associated with Kruskal-Wallis tests comparing tree density, fly herbivory, and wasp parasitism rates across fine grain landcover categories.
