# Supplementary material for: Urban Land Use Decouples Plant-Herbivore-Parasitoid Interactions at Multiple Spatial Scales
Source: PLoS One. 2014 Jul 14;9(7):e102127. doi: 10.1371/journal.pone.0102127 (PMC4096920; doi:10.1371/journal.pone.0102127)
Supplement: Table S6 — Top five regression models in which landcover proportions predicted herbivory and parasitism rates. Results are shown at the level of the entire landscape and among urban/suburban and agricultural subsets of sites. Summary statistics for individual models are shown. Each model is designated with a number corresponding to the set of coefficients and p-values associated with individual independent variables within the model as shown in the lower half of the table. Statistics are not shown for urban/suburban models associated with walnut flies (R. suavis) because none of these models were significant. EL = entire landscape, AG = agricultural site subset, URB = urban/suburban site subset. (DOCX) [file pone.0102127.s010.docx]

**Table S6.** **Top five regression models in which landcover proportions predicted herbivory and parasitism rates.** Results are shown at the level of the entire landscape and among urban/ suburban and agricultural subsets of sites. Summary statistics for individual models are shown. Each model is designated with a number corresponding to the set of coefficients and p-values associated with individual independent variables within the model as shown in the lower half of the table. Statistics are not shown for urban/ suburban models associated with walnut flies (*R. suavis*) because none of these models were significant. EL= entire landscape, AG= agricultural site subset, URB= urban/ suburban site subset.

| **Dependent variable** | **Subset** | **MODEL #** | **Independent variables included** | | **Extent (radius)** | **Adj. R2** | **R2** | **P** | **F** | **AIC** | **LL** | **N** |
| --- | --- | --- | --- | --- | --- | --- | --- | --- | --- | --- | --- | --- |
| *R. suavis* larvae per *J. nigra* fruits | EL | 1 | IPC/ LDD/ wooded/ water | | 3 km | 0.149 | 0.213 | 0.017 | 3.322 | 178.162 | -84.081 | 54 |
|  | EL | 2 | IPC/ LDD/ wooded | | 3 km | 0.148 | 0.196 | 0.012 | 4.073 | 177.315 | -84.658 | 54 |
|  | EL | 3 | IPC/ LDD/ wooded/ WPC | | 3 km | 0.138 | 0.203 | 0.023 | 3.116 | 178.881 | -84.441 | 54 |
|  | EL | 4 | IPC/ LDD/ wooded/ MDD | | 3 km | 0.137 | 0.202 | 0.023 | 3.11 | 178.903 | -84.451 | 54 |
|  | EL | 5 | IPC/ LDD/ wooded/ herbaceous | | 3 km | 0.137 | 0.202 | 0.023 | 3.11 | 178.919 | -84.46 | 54 |
|  | AG | 6 | Wooded/ HDD/ MDD/ LDD/ OD/ water | | 2 km | 0.385 | 0.533 | 0.015 | 3.613 | 78.3439 | -32.172 | 25 |
|  | AG | 7 | Wooded/ HDD/ MDD/ LDD/ OD/ water/WPC | | 2 km | 0.357 | 0.537 | 0.029 | 2.985 | 80.105 | -32.052 | 25 |
|  | AG | 8 | Wooded/ HDD/ MDD/ LDD/ OD/ water/ IPC | | 2 km | 0.355 | 0.536 | 0.03 | 2.967 | 80.185 | -32.093 | 25 |
|  | AG | 9 | Wooded/ HDD/ MDD/ LDD/ OD | | 2 km | 0.333 | 0.466 | 0.02 | 3.493 | 87.363 | -33.907 | 25 |
|  | AG | 10 | Wooded/ HDD/ MDD/ LDD/ OD/ water/ WPC/ IPC | | 2 km | 0.329 | 0.544 | 0.051 | 2.53 | 81.745 | -31.872 | 25 |
|  | URB | NA | HDD/ LDD | | 500 m | 0.118 | 0.198 | 0.11 | 2.468 | 74.236 | -34.118 | 23 |
|  | URB | NA | HDD/ LDD/ wooded/ herbaceous | | 500 m | 0.114 | 0.275 | 0.191 | 1.711 | 75.898 | -32.949 | 23 |
|  | URB | NA | HDD/ LDD/ wooded | | 500 m | 0.114 | 0.235 | 0.157 | 1.943 | 75.155 | -33.577 | 23 |
|  | URB | NA | HDD/ LDD/ wooded/ OD | | 500 m | 0.093 | 0.258 | 0.227 | 1.562 | 76.455 | -33.228 | 23 |
|  | URB | NA | HDD/ LDD/ water | | 500 m | 0.092 | 0.216 | 0.193 | 1.741 | 75.721 | -33.861 | 23 |
|  |  |  |  |  |  |  |  |  |  |  |  |  |
| *R. cingulata* larvae per *P. serotina* fruits | EL | 11 | IPC/ WPC/ water/ HDD/ wooded | | 4 km | 0.292 | 0.38 | 0.004 | 4.295 | 66.273 | -27.136 | 42 |
|  | EL | 12 | WPC/ water/ HDD/ wooded | | 4 km | 0.286 | 0.357 | 0.003 | 4.997 | 65.782 | -27.891 | 42 |
| EL | 13 | IPC/ WPC/ water/ HDD/ wooded/herbaceous | | 4 km | 0.274 | 0.383 | 0.008 | 3.517 | 68.094 | -27.047 | 42 |  |
| EL | 14 | IPC/ WPC/ water/ HDD | | 4 km | 0.269 | 0.343 | 0.004 | 4.689 | 66.696 | -28.348 | 42 |  |
| EL | 15 | water/ HDD/ wooded | | 4 km | 0.267 | 0.322 | 0.002 | 5.868 | 65.932 | -28.966 | 42 |  |
| AG | 16 | IPC/ water/ LDD | | 250 m | 0.394 | 0.481 | 0.007 | 5.561 | 21.646 | -6.823 | 23 |  |
| AG | 17 | IPC/ water/ LDD/ OD | | 250 m | 0.376 | 0.494 | 0.016 | 4.165 | 23.047 | -6.523 | 23 |  |
| AG | 18 | IPC/ water/ LDD/ HDD | | 250 m | 0.367 | 0.488 | 0.017 | 4.047 | 23.357 | -6.679 | 23 |  |
| AG | 19 | IPC/ water/ LDD/ MDD | | 250 m | 0.362 | 0.483 | 0.019 | 3.98 | 23.541 | -6.77 | 23 |  |
| AG | 20 | IPC/ water/ LDD/ WPC | | 250 m | 0.359 | 0.481 | 0.019 | 3.94 | 23.644 | -6.822 | 23 |  |
| URB | 21 | IPC/ WPC/ water/ HDD/ MDD/ wooded | | 250 m | 0.813 | 0.899 | 0.003 | 10.424 | -14.067 | 14.033 | 14 |  |
| URB | 22 | IPC/ WPC/ water/ HDD/ MDD/ woody/herbaceous | | 250 m | 0.797 | 0.906 | 0.01 | 8.3 | -13.084 | 14.542 | 14 |  |
| URB | 23 | IPC/ WPC/ water/ HDD/ wooded/ herbaceous | | 250 m | 0.796 | 0.89 | 0.005 | 9.448 | -12.874 | 13.437 | 14 |  |
| URB | 24 | IPC/ WPC/ water/ HDD/ wooded | | 250 m | 0.794 | 0.873 | 0.002 | 11.031 | -12.848 | 12.424 | 14 |  |
| URB | 25 | IPC/ WPC/ HDD/ MDD/ wooded | | 250 m | 0.786 | 0.868 | 0.002 | 10.562 | -12.318 | 12.159 | 14 |  |
|  |  |  |  |  |  |  |  |  |  |  |  |  |
| *D. ferrugineum* wasps per *R. cingulata* larvae | EL | 26 | IPC/ MDD/ wooded/ HDD/ water | | 250 m | 0.543 | 0.647 | 0.002 | 6.229 | 46.775 | -17.387 | 24 |
|  | EL | 27 | IPC/ MDD/ wooded | | 250 m | 0.535 | 0.599 | 0.0005 | 9.453 | 45.255 | -18.856 | 24 |
|  | EL | 28 | IPC/ MDD/ wooded/ LDD | | 250 m | 0.525 | 0.612 | 0.001 | 7.091 | 46.957 | -18.479 | 24 |
|  | EL | 29 | IPC/ MDD/ wooded/ HDD/ water/ LDD | | 250 m | 0.521 | 0.652 | 0.005 | 4.996 | 48.442 | -17.221 | 24 |
|  | EL | 30 | IPC/ MDD/ wooded/ HDD/ water/ WPC | | 250 m | 0.518 | 0.65 | 0.005 | 4.946 | 48.593 | -17.297 | 24 |

| **INDIVIDUAL COEFFICIENTS AND SIGNIFICANCE BY MODEL # (see numbers above)** | | | | |
| --- | --- | --- | --- | --- |
| **MODEL #** | **Variable** | **Coefficients** | **T-statistic** | **P-value** |
| 1 | IPC | -10.196 | -1.306 | 0.198 |
|  | LDD | -12.823 | -2.117 | 0.039 |
|  | WOODED | 10.678 | 1.028 | 0.309 |
|  | WATER | -8.502 | -2.923 | 0.005 |
|  |  |  |  |  |
| 2 | IPC | -13.931 | -2.014 | 0.049 |
|  | LDD | -14.959 | -2.628 | 0.011 |
|  | WOODED | -8.831 | -3.053 | 0.004 |
|  |  |  |  |  |
| 3 | IPC | -17.099 | -1.991 | 0.052 |
|  | LDD | -14.194 | -2.424 | 0.019 |
|  | WOODED | -8.096 | -2.582 | 0.013 |
|  | WPC | 2.604 | 0.629 | 0.533 |
|  |  |  |  |  |
| 4 | IPC | -19.468 | -1.706 | 0.094 |
|  | LDD | -15.089 | -2.632 | 0.011 |
|  | WOODED | -11.206 | -2.312 | 0.025 |
|  | MDD | -9.404 | -0.613 | 0.543 |
|  |  |  |  |  |
| 5 | IPC | -13.782 | -1.978 | 0.054 |
|  | LDD | -16.091 | -2.668 | 0.011 |
|  | WOODED | -8.749 | -3.002 | 0.004 |
|  | HERB. | -2.363 | -0.601 | 0.552 |
|  |  |  |  |  |
| 6 | WOODED | -2.073 | -1.341 | 0.196 |
|  | HDD | 30.115 | 1.230 | 0.234 |
|  | MDD | -286.535 | -3.001 | 0.007 |
|  | LDD | 223.513 | 2.805 | 0.011 |
|  | OD | -77.941 | -2.277 | 0.035 |
|  | WATER | 51.629 | 1.647 | 0.116 |
|  |  |  |  |  |
| 7 | WOODED | -0.781 | -0.221 | 0.828 |
|  | HDD | 33.254 | 1.270 | 0.220 |
|  | MDD | -297.972 | -2.933 | 0.009 |
|  | LDD | 234.675 | 2.730 | 0.014 |
|  | OD | -81.446 | -2.260 | 0.036 |
|  | WATER | 50.745 | 1.580 | 0.132 |
|  | WPC | 1.507 | 0.408 | 0.688 |
|  |  |  |  |  |
| 8 | WOODED | -2.958 | -0.954 | 0.353 |
|  | HDD | 29.339 | 1.165 | 0.259 |
|  | MDD | -282.816 | -2.873 | 0.010 |
|  | LDD | 215.638 | 2.537 | 0.021 |
|  | OD | -73.448 | -1.954 | 0.066 |
|  | WATER | 48.050 | 1.419 | 0.173 |
|  | IPC | -2.347 | -0.332 | 0.744 |
|  |  |  |  |  |
| 9 | WOODED | -2.941 | -1.942 | 0.066 |
|  | HDD | 30.783 | 1.207 | 0.242 |
|  | MDD | -193.365 | -2.413 | 0.026 |
|  | LDD | 144.266 | 2.179 | 0.041 |
|  | OD | -45.262 | -1.557 | 0.135 |
|  |  |  |  |  |
| 10 | WOODED | -1.626 | -0.405 | 0.690 |
|  | HDD | 33.379 | 1.247 | 0.229 |
|  | MDD | -297.012 | -2.861 | 0.011 |
|  | LDD | 226.997 | 2.543 | 0.021 |
|  | OD | -75.828 | -1.965 | 0.066 |
|  | WATER | 44.681 | 1.272 | 0.220 |
|  | WPC | 2.159 | 0.539 | 0.597 |
|  | IPC | -3.726 | -0.487 | 0.633 |
|  |  |  |  |  |
| 11 | IPC | 5.710 | 1.145 | 0.260 |
|  | WPC | -4.946 | -1.801 | 0.080 |
|  | WATER | 30.231 | 3.462 | 0.001 |
|  | HDD | -17.719 | -1.708 | 0.097 |
|  | WOODED | -2.825 | -1.460 | 0.153 |
|  |  |  |  |  |
| 12 | WPC | -2.749 | -1.392 | 0.172 |
|  | WATER | 26.419 | 3.258 | 0.002 |
|  | HDD | -23.872 | -2.678 | 0.011 |
|  | WOODED | -3.469 | -1.865 | 0.070 |
|  |  |  |  |  |
| 13 | IPC | 6.679 | 1.184 | 0.244 |
|  | WPC | -5.522 | -1.749 | 0.089 |
|  | WATER | 28.355 | 2.809 | 0.008 |
|  | HDD | -19.395 | -1.706 | 0.097 |
|  | WOODED | -2.738 | -1.388 | 0.174 |
|  | HERB. | -1.431 | -0.385 | 0.702 |
|  |  |  |  |  |
| 14 | IPC | 7.822 | 1.615 | 0.115 |
|  | WPC | -3.573 | -1.363 | 0.181 |
|  | WATER | 25.592 | 3.098 | 0.004 |
|  | HDD | -4.303 | -0.880 | 0.385 |
|  |  |  |  |  |
| 15 | WATER | 25.323 | 3.098 | 0.004 |
|  | HDD | -12.813 | -3.127 | 0.003 |
|  | WOODED | -1.408 | -1.235 | 0.225 |
|  |  |  |  |  |
| 16 | IPC | 1.596 | 2.351 | 0.030 |
|  | WATER | -59.817 | -3.842 | 0.001 |
|  | LDD | -7.955 | -2.315 | 0.033 |
|  |  |  |  |  |
| 17 | IPC | 1.773 | 2.409 | 0.028 |
|  | WATER | -63.743 | -3.791 | 0.001 |
|  | LDD | -11.183 | -1.907 | 0.074 |
|  | OD | 1.172 | 0.685 | 0.503 |
|  |  |  |  |  |
| 18 | IPC | 1.677 | 2.346 | 0.031 |
|  | WATER | -63.403 | -3.598 | 0.002 |
|  | LDD | -9.214 | -2.092 | 0.052 |
|  | HDD | 2.312 | 0.474 | 0.642 |
|  |  |  |  |  |
| 19 | IPC | 1.698 | 2.168 | 0.045 |
|  | WATER | -61.305 | -3.646 | 0.002 |
|  | LDD | -9.133 | -1.682 | 0.111 |
|  | MDD | 1.622 | 0.285 | 0.779 |
|  |  |  |  |  |
| 20 | IPC | 1.605 | 2.181 | 0.044 |
|  | WATER | -59.997 | -3.615 | 0.002 |
|  | LDD | -7.946 | -2.243 | 0.039 |
|  | WPC | 0.015 | 0.042 | 0.967 |
|  |  |  |  |  |
| 21 | IPC | 21.851 | 6.046 | 0.001 |
|  | WPC | -164.225 | -5.589 | 0.001 |
|  | WATER | 4.027 | 1.466 | 0.186 |
|  | HDD | -4.554 | -1.924 | 0.096 |
|  | MDD | 0.974 | 1.608 | 0.152 |
|  | WOODED | 0.631 | 3.453 | 0.011 |
|  |  |  |  |  |
| 22 | IPC | 24.853 | 4.255 | 0.005 |
|  | WPC | -176.551 | -4.949 | 0.003 |
|  | WATER | 3.549 | 1.204 | 0.274 |
|  | HDD | -4.161 | -1.643 | 0.152 |
|  | MDD | 0.939 | 1.483 | 0.189 |
|  | WOODED | 0.724 | 3.077 | 0.022 |
|  | HERB. | -0.220 | -0.672 | 0.526 |
|  |  |  |  |  |
| 23 | IPC | 5.554 | 2.216 | 0.062 |
|  | WPC | -17.406 | -1.453 | 0.190 |
|  | WATER | 7.588 | 5.351 | 0.001 |
|  | HDD | -2.741 | -3.972 | 0.005 |
|  | WOODED | 0.510 | 1.844 | 0.108 |
|  | HERB. | -0.486 | -1.044 | 0.331 |
|  |  |  |  |  |
| 24 | IPC | 4.231 | 1.946 | 0.088 |
|  | WPC | -23.336 | -2.200 | 0.059 |
|  | WATER | 6.870 | 5.509 | 0.001 |
|  | HDD | -2.865 | -4.190 | 0.003 |
|  | WOODED | 0.361 | 1.515 | 0.168 |
|  |  |  |  |  |
| 25 | IPC | 22.208 | 5.759 | 0.000 |
|  | WPC | -165.816 | -5.281 | 0.001 |
|  | HDD | -1.412 | -1.315 | 0.225 |
|  | MDD | 1.371 | 2.364 | 0.046 |
|  | WOODED | 0.618 | 3.165 | 0.013 |
|  |  |  |  |  |
| 26 | IPC | 5.589 | 3.972 | 0.001 |
|  | MDD | 4.033 | 0.969 | 0.346 |
|  | WOODED | 1.894 | 3.018 | 0.008 |
|  | HDD | -52.751 | -1.761 | 0.096 |
|  | WATER | 58.281 | 1.719 | 0.104 |
|  |  |  |  |  |
| 27 | IPC | 6.043 | 4.297 | 0.000 |
|  | MDD | 9.342 | 3.397 | 0.003 |
|  | WOODED | 2.012 | 3.189 | 0.005 |
|  |  |  |  |  |
| 28 | IPC | 5.715 | 3.848 | 0.001 |
|  | MDD | 12.070 | 2.670 | 0.016 |
|  | WOODED | 1.914 | 2.940 | 0.009 |
|  | LDD | -3.292 | -0.765 | 0.454 |
|  |  |  |  |  |
| 29 | IPC | 5.467 | 3.712 | 0.002 |
|  | MDD | 5.930 | 0.945 | 0.359 |
|  | WOODED | 1.870 | 2.893 | 0.011 |
|  | HDD | -49.127 | -1.538 | 0.144 |
|  | WATER | 55.920 | 1.587 | 0.132 |
|  | LDD | -2.150 | -0.412 | 0.686 |
|  |  |  |  |  |
| 30 | IPC | 5.736 | 3.786 | 0.002 |
|  | MDD | 4.190 | 0.974 | 0.345 |
|  | WOODED | 2.005 | 2.746 | 0.014 |
|  | HDD | -53.215 | -1.728 | 0.103 |
|  | WATER | 59.280 | 1.695 | 0.109 |
|  | WPC | 0.246 | 0.325 | 0.749 |
