# Supplementary material for: Urban Land Use Decouples Plant-Herbivore-Parasitoid Interactions at Multiple Spatial Scales
Source: PLoS One. 2014 Jul 14;9(7):e102127. doi: 10.1371/journal.pone.0102127 (PMC4096920; doi:10.1371/journal.pone.0102127)
Supplement: Table S7 — Significant main effects of landcover categories on herbivore densities. Results are shown for significant main effects found in generalized linear models designed to assess responses of insect densities to fine versus coarse landcover categories, and potential interactions between the two grains. Direction of effect is indicated for significant parameters. A. Main effects of landcover category on walnut fly density. B. Main effects of landcover category on cherry fly density. (DOCX) [file pone.0102127.s011.docx]

**Table S7.**

| A.WALNUT FLIES (N= 54) | | **Wald Chi-Square** | **p** | **df** | **Effect** |
| --- | --- | --- | --- | --- | --- |
| **Factors** (Main effects) | Fine grain | 32.423 | < 0.001 | 6 |  |
|  | Coarse grain | 1.920 | 0.383 | 2 |  |
|  | Fine x coarse interaction | 5.779 | 0.123 | 3 |  |
|  |  |  |  |  |  |
| **Significant parameters** | OD (Fine) | 11.866 | 0.001 | 1 | negative |
|  | LDD (Fine) | 5.366 | 0.021 | 1 | negative |
|  | MDD (Fine) | 13.017 | < 0.001 | 1 | negative |
|  | HDD (Fine) | 3.751 | 0.053 | 1 | negative |

| B. CHERRY FLIES (N= 42) | | **Wald Chi-Square** | **p** | **df** | **Effect** |
| --- | --- | --- | --- | --- | --- |
| **Factors** (Main effects) | Fine grain | 6.013 | 0.305 | 5 |  |
|  | Coarse grain | 13.261 | 0.001 | 2 |  |
|  | Fine x coarse interaction | 8.631 | 0.013 | 2 |  |
|  |  |  |  |  |  |
| **Significant parameters** | Crop (Fine) | 6.546 | 0.011 | 1 | negative |
|  | Herbaceous (fine) | 6.073 | 0.014 | 1 | positive |
|  | Herbaceous (fine) x Urban/ Suburban (coarse) interaction | 8.516 | 0.004 | 1 | negative |
|  | Herbaceous (fine) x Agricultural (coarse) interaction | 5.073 | 0.024 | 1 | negative |

**Table S7. Significant main effects of landcover categories on herbivore densities.** Results are shown for significant main effects found in generalized linear models designed to assess responses of insect densities to fine versus coarse landcover categories, and potential interactions between the two grains. Direction of effect is indicated for significant parameters. A. Main effects of landcover category on walnut fly density. B. Main effects of landcover category on cherry fly density.
