# Supplementary material for: Urban Land Use Decouples Plant-Herbivore-Parasitoid Interactions at Multiple Spatial Scales
Source: PLoS One. 2014 Jul 14;9(7):e102127. doi: 10.1371/journal.pone.0102127 (PMC4096920; doi:10.1371/journal.pone.0102127)
Supplement: Table S8 — Significant main effects of landcover proportions on densities in cherry-associated insects. Results are shown for significant main effects found in generalized linear models designed to assess responses of insect densities to local (250 m) versus larger (4 km) proportions of individual landcover variables, and potential interactions between the two buffer distances. Direction of effect is indicated for significant main effects. A. Main effects of the proportion of open water on cherry fly density. Results for models within agricultural and urban/suburban subsets of sites are also shown. B. Main effects of the proportion of total pooled urban landcover on cherry wasp density. (DOCX) [file pone.0102127.s012.docx]

**Table S8.**

| A. | **Covariates (Main effects)** | **Wald Chi-square** | **p** | **df** | **Effect** |
| --- | --- | --- | --- | --- | --- |
| **Entire landscape** | Water (250 m radius) | 0.364 | 0.546 | 1 |  |
| (N= 42) | Water (4 km radius) | 4.024 | 0.045 | 1 | positive |
|  | Interaction (250 m x 4 km) | 0.113 | 0.737 | 1 |  |
|  |  |  |  |  |  |
| **Agriculture** | Water (250 m radius) | 4.907 | 0.027 | 1 | negative |
| (N= 24) | Water (4 km radius) | 0.726 | 0.394 | 1 |  |
|  | Interaction (250 m x 4 km) | 2.357 | 0.125 | 1 |  |
|  |  |  |  |  |  |
| **Urban/ Suburban** | Water (250 m radius) | 29.264 | < 0.001 | 1 | negative |
| (N= 14) | Water (4 km radius) | 0.293 | 0.588 |  |  |
|  | Interaction (250 m x 4 km) | 29.265 | < 0.001 | 1 | positive |

| B. | **Covariates (Main effects)** | **Wald Chi-square** | **p** | **df** | **Effect** |
| --- | --- | --- | --- | --- | --- |
| **Entire landscape** | Urban (250 m radius) | 6.684 | 0.010 | 1 | negative |
| (N= 24) | Urban (4 km radius) | 2.000 | 0.157 | 1 |  |
|  | Interaction (250 m x 4 km) | 8.625 | 0.003 | 1 | negative |

**Table S8. Significant main effects of landcover proportions on densities in cherry-associated insects.** Results are shown for significant main effects found in generalized linear models designed to assess responses of insect densities to local (250 m) versus larger (4 km) proportions of individual landcover variables, and potential interactions between the two buffer distances. Direction of effect is indicated for significant main effects. A. Main effects of the proportion of open water on cherry fly density. Results for models within agricultural and urban/ suburban subsets of sites are also shown. B. Main effects of the proportion of total pooled urban landcover on cherry wasp density.
